# Systematic discovery of the functional impact of somatic genome alterations in individual tumors through tumor-specific causal inference

**DOI:** 10.1371/journal.pcbi.1007088

**Published:** 2019-07-05

**Authors:** Chunhui Cai, Gregory F. Cooper, Kevin N. Lu, Xiaojun Ma, Shuping Xu, Zhenlong Zhao, Xueer Chen, Yifan Xue, Adrian V. Lee, Nathan Clark, Vicky Chen, Songjian Lu, Lujia Chen, Liyue Yu, Harry S. Hochheiser, Xia Jiang, Q. Jane Wang, Xinghua Lu

**Affiliations:** 1 Department of Biomedical Informatics, School of Medicine, University of Pittsburgh, Pittsburgh, PA, United States of America; 2 Center for Causal Discovery, Pittsburgh, PA, United States of America; 3 Department of Pharmacology and Chemical Biology, University of Pittsburgh, Pittsburgh, PA, United States of America; 4 Magee Women’s Cancer Research Center, Pittsburgh, PA, United States of America; 5 UPMC Hillman Cancer Center, University of Pittsburgh Medical Center, Pittsburgh, PA, United States of America; 6 Department of Computational Biology and Systems Biology, School of Medicine, University of Pittsburgh, Pittsburgh, PA, United States of America; MUSC, UNITED STATES

## Abstract

Cancer is mainly caused by somatic genome alterations (SGAs). Precision oncology involves identifying and targeting tumor-specific aberrations resulting from causative SGAs. We developed a novel tumor-specific computational framework that finds the likely causative SGAs in an individual tumor and estimates their impact on oncogenic processes, which suggests the disease mechanisms that are acting in that tumor. This information can be used to guide precision oncology. We report a tumor-specific causal inference (TCI) framework, which estimates causative SGAs by modeling causal relationships between SGAs and molecular phenotypes (e.g., transcriptomic, proteomic, or metabolomic changes) within an individual tumor. We applied the TCI algorithm to tumors from The Cancer Genome Atlas (TCGA) and estimated for each tumor the SGAs that causally regulate the differentially expressed genes (DEGs) in that tumor. Overall, TCI identified 634 SGAs that are predicted to cause cancer-related DEGs in a significant number of tumors, including most of the previously known drivers and many novel candidate cancer drivers. The inferred causal relationships are statistically robust and biologically sensible, and multiple lines of experimental evidence support the predicted functional impact of both the well-known and the novel candidate drivers that are predicted by TCI. TCI provides a unified framework that integrates multiple types of SGAs and molecular phenotypes to estimate which genome perturbations are causally influencing one or more molecular/cellular phenotypes in an individual tumor. By identifying major candidate drivers and revealing their functional impact in an individual tumor, TCI sheds light on the disease mechanisms of that tumor, which can serve to advance our basic knowledge of cancer biology and to support precision oncology that provides tailored treatment of individual tumors.

## Author summary

Precision oncology relies on the capability of identifying and targeting tumor-specific aberrations resulting from causative genomic alterations in each tumor. Conventional cancer driver identification methods identify candidate cancer driver genes as those exhibit an alteration frequency significantly above the expected frequency that would occur by random chance in a population of tumor samples. This population-based nature prevents them from performing instance-specific discovery, and alteration frequency does not contain information regarding the functional impact of candidate driver genes identified in this approach. Here, we report a novel Bayesian causal discovery framework, referred to as tumor-specific causal inference (TCI), which identifies candidate driver genes as the ones that bear significant functional impact on cancer-related molecular phenotypes at the individual tumor level. By discovering candidate drivers and their function impact in each individual tumor, TCI analysis reveals information that is of value for both general cancer biology research and precision oncology.

## Introduction

Cancer is mainly caused by a variety of SGAs, including, but not limited to, somatic mutations (SMs) [1, 2], somatic DNA copy number alterations (SCNAs) [3, 4], chromosome structure variations [5–7], and epigenetic changes [8–10]. Each tumor hosts a unique combination of SGAs ranging in number from hundreds to thousands, of which only a small fraction contributes to tumorigenesis (drivers), while the rest are non-consequential (passengers). Identifying causative SGAs that underlie different oncogenic processes [11], such as metastasis or immune evasion, in an individual tumor is of fundamental importance in cancer biology and precision oncology [12–14].

Current methods for identifying cancer driver genes concentrate on finding those that have a higher than expected mutation rate in a cohort of tumor samples [15–17]. Some methods focus on specific mutation sites (e.g., mutation hotspots at specific amino acids or within the 3D functional domain of a protein) that likely affect the function of those proteins encoded by the mutant genes [17–22]. These mutation-centric, frequency-based models have successfully identified many major oncogenes and tumor suppressors across cancer types. However, they do not directly determine the functional impact of mutations, because mutation frequency of a gene (either at the gene or at the specific amino acid level) does not directly reflect which molecular or cellular processes will be affected by the altered gene product.

Besides mutations, other SGA events affecting driver genes also contribute to cancer development, such as SCNAs [3, 4, 23], chromosome structure variation [5–7], and epigenetic changes [8–10]. Currently, analyses of SMs, SCNAs, structure variation, and epigenetic data are usually carried out separately, with distinct statistical models for different types of data [1, 2, 16, 24, 25]. Such disconnection is largely due to the lack of a unifying statistical framework that is able to integrate diverse data. Integrating diverse data can provide increased statistical power to detect biological function and to gain biological insights by pooling diverse information to assess the role of a driver gene in oncogenesis. A Bayesian approach has the potential to provide such a unifying framework.

Some recent studies have started to employ a Bayesian framework to infer relationships between cancer driver mutations and other omics changes, such as transcriptomic changes. Razi et al. proposed a hybrid Bayesian method to capture the non-linear regulatory effects on the gene expression levels based on a predefined signaling network [26]. The iDriver is another non-parametric Bayesian framework developed by Yang et al. which models the joint distribution of multiomics data and identified 45 novel driver genes that showed significant deviations from the background in at least one omics data [27]. The above methods employ a Bayesian approach to estimate model parameters, rather than searching for causal networks, which is the focus of the current paper. More recently, Wang et al. developed a Bayesian (regularized) regression model, referred to as rDriver, to model the relationships between mutations and gene expression changes [28]. However, it is a population-based regression method, which does not take into account the tumor-specific changes.

In this study, we designed a general framework based on Bayesian causal modeling and discovery [29–31] that estimates the causal relationships between SGAs and molecular phenotypes observed in an individual tumor [32]. We call it the Tumor-specific Causal Inference (TCI) method. By being Bayesian in design, TCI is flexible in the types of data that define both the SGAs and the molecular phenotypes. By being tumor-specific, TCI is able to model the functional causal relationships between the SGAs and the molecular phenotypes *in a given tumor*. The tumor-specific nature of the TCI differentiates it from previous methods that aim to detect the association between genomic variations and quantitative traits, in particular, the expression quantitative trait loci (eQTL) analysis, which is a population-based method that requires a large number of cases to estimate associations between SGAs and molecular phenotypes across a population of tumors [33, 34]. However, eQTL does not predict the causal influence of SGAs on molecular phenotypes in a given tumor. Identification of SGAs that have a specific functional impact on molecular phenotypes in an individual tumor can help to differentiate candidate driver SGAs from passengers and shed light on the disease mechanism of that tumor, which could guide precision treatment of the tumor.

## Results

### TCI is an integrative framework for discovering the functional impact of SGAs in an individual tumor

**Fig 1:**
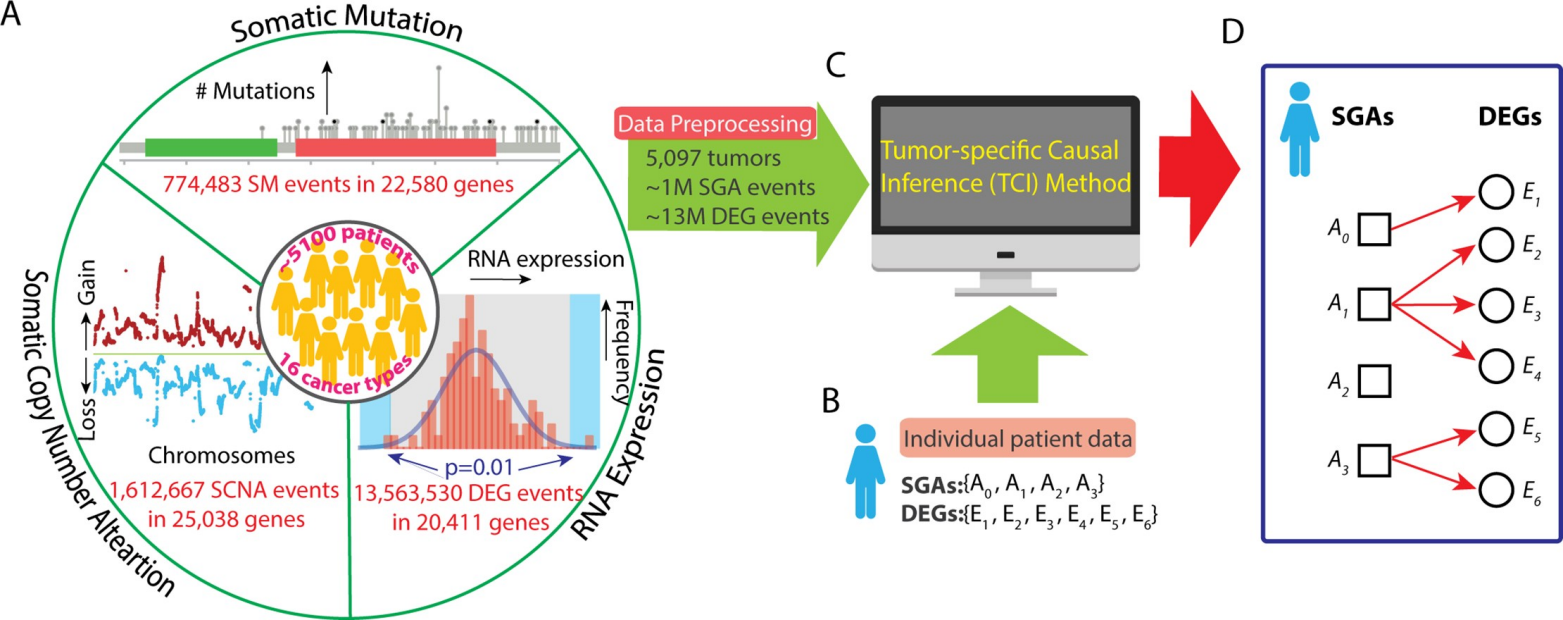
Workflow of TCI analysis. **A.** A compendium of cancer omics data is used as the training dataset. Three types of data from the 5,097 pan-cancer tumors were used in this study, including SM data (774,483 mutation events in 22,580 genes), SCNA data (1,612,667 copy number alteration events in 25,038 genes), and gene expression data (13,563,530 DEG events in 20,411 genes). SM and SCNA data were integrated as SGA data. Expression of each gene in each tumor was compared to a distribution of the same gene in the “normal control” samples, and, if a gene’s expression value was outside the significance boundary, it was designated as a DEG in the tumor. The final dataset included 5,097 tumors with 1,364,207 SGA events and 13,549,660 DEG events. **B.** A set of SGAs and a set of DEGs from an individual tumor as input for TCI modeling. **C.** The TCI algorithm infers the causal relationships between SGAs and DEGs for a given tumor *t* and output a tumor-specific causal model. **D.** A hypothetic model illustrates the results of TCI analysis. In this tumor, *SGA_SET*_*t*_ has three SGAs plus the non-specific factor *A*_0_, and *DEG_SET*_*t*_ has six DEG variables. Each *E*_*i*_ must have exactly one arc into it, which represents having one cause among the variables in *SGA_SET*_*t*_. In this model, *E*_1_ is caused by *A*_0_; *E*_2_, *E*_3_, E_4_ are caused by *A*_1_; *E*_5_, *E*_6_ are caused by *A*_3_; *A*_2_ does not have any regulatory impact.

We designed the TCI algorithm to discover the causal relationships between SGAs and DEGs observed in an individual tumor. Specifically, given a tumor *t* hosting a set of SGAs (*SGA_SET*_*t*_) and a set of DEGs (*DEG_SET*_*t*_), TCI estimates the causal relationships between SGAs and DEGs using a bipartite causal Bayesian network [29–31] (Fig 1). It searches for the tumor-specific causal model *M*_*t*_ with a maximal posterior probability *P*(*M*_*t*_|*D*) given the dataset *D* (containing SGAs and DEGs). The tumor-specific nature of the TCI model is reflected by the assumption that a molecular phenotype change (e.g., a DEG) observed in a specific tumor should be attributed with a high probability to one of the SGAs observed in the tumor that explains the phenotype well in the dataset *D*, or alternatively, to a non-specific cause denoted as *A*_*0*_ that collectively represents unmeasured genomic events or non-SGA causes, such as the tumor microenvironment (Detailed description of TCI method is included in S1 Text).

Although the indices of the SGAs and DEGs for the patient case shown are sequential, but in general they would indicate different SGAs and DEGs in different tumors.

TCI achieves tumor-specific causal discovery through several innovative approaches. Consider a collection of genomic data (denoted as *D*) from TCGA, and the data from a new tumor *t* hosting a set of SGAs (*SGA_SET*_*t*_) and a set of DEGs (*DEG_SET*_*t*_). For a DEG event *E*_*i*_ among the *DEG_SET*_*t*_, TCI aims to identify an SGA *A*_*h*_ among the *SGA_SET*_*t*_ that most likely caused *E*_*i*_, or alternatively, TCI may assign the factor *A*_*0*_ as a non-specific cause. TCI evaluates the posterior probability that *A*_*h*_ causes *E*_*i*_, which we denote as *A*_*h*_→*E*_*i*_, using a Bayesian framework as follows:

$$P(A_{h}\to E_{i}|D)=\frac{1}{Z}P(A_{h}\to E_{i})P(D|A_{h}\to E_{i}),$$ (1)

where

$$Z=\sum_{j=0}^{\left|SGA\_SET_{t}\right|}P(A_{j}\to E_{i})P(D|A_{j}\to E_{i})$$ (2)

is a normalization term. From the above equations, one can see that a potential causal SGA *A*_*h*_ only competes with other SGAs observed in the same tumor to explain a molecular phenotype *E*_*i*_. This allows a less frequent SGA (*A*_*h*_) to be assigned with high posterior probability as the cause for a changed phenotype (*E*_*i*_) in a specific tumor, as long as *A*_*h*_ is the most plausible cause when compared with the other SGAs in the same tumor. TCI involves the two terms on the right of the Eq 1: the *prior probability* that *A*_*h*_ causes *E*_*i*_, namely, *P*(*A*_*h*_→*E*_*i*_), which can be evaluated at a population-level prior to observing current tumor *t*, and the conditional probability (aka the *marginal likelihood*) of data *D*, *P*(*D*|*A*_*h*_→*E*_*i*_), given that *A*_*h*_→*E*_*i*_, which assesses the functional impact of the causal edges (Supplementary method in S1 Text). This approach allows TCI to integrate useful aspects of a frequency-oriented framework (via the prior probability) and a cellular-function-oriented framework (via the marginal likelihood). An important innovation of TCI is the procedure for evaluating *P*(*D*|*A*_*h*_→*E*_*i*_), which consist of assessing how well *A*_*h*_ explains the variance of *E*_*i*_ in tumors hosting *A*_*h*_ (aka, “tumors like me”), as well as how well the variance of *E*_*i*_ is explained in tumors do not host *A*_*h*_. Finally, depending on the composition of *SGA_SET*_*t*_, the tumor-specific prior probability *P*(*A*_*h*_→*E*_*i*_) for the same causal edge between *A*_*h*_ and *E*_*i*_ can be different in different tumors, and therefore tumor-specific (see the Materials and methods section for details).

**Fig 2:**
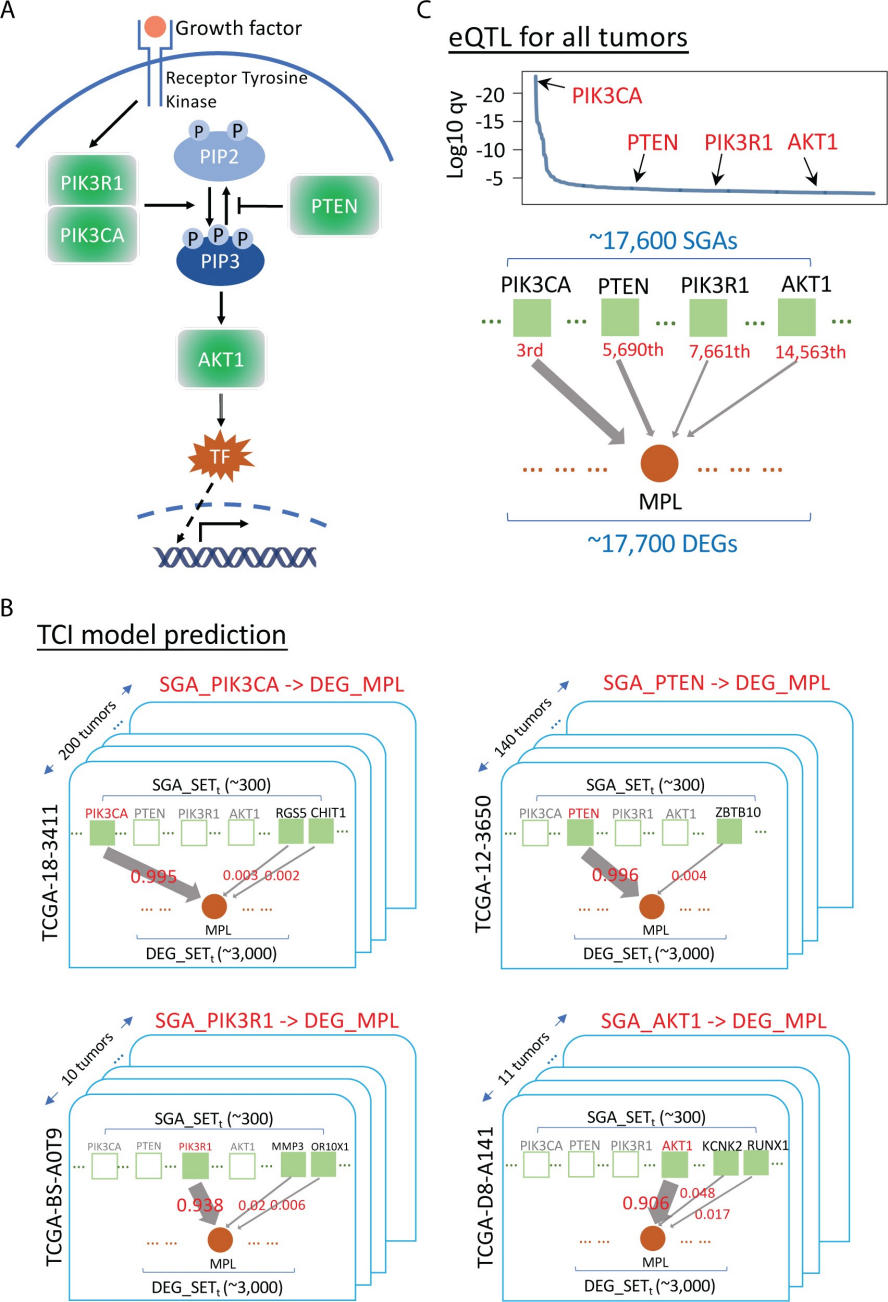
Estimation of the most probable causative SGAs for *MPL* by TCI and eQTL. **A.** A diagram of PI3K/AKT pathway, with *PIK3CA*, *PTEN*, *PIK3R1* and *AKT1* as key signaling proteins in the pathway. **B.** Results of TCI analysis of the most probable causes of the DEG *MPL*. There are ~300 SGAs and ~3,000 DEGs in each tumor on average, which are organized as a bipartite graph respectively. Solid green squares represent SGAs present in the current tumor; empty green square represent SGAs not present in the current tumor. For a DEG observed in a tumor, e.g., *MPL*, TCI aims to search for the most probable cause among SGAs observed in the tumor. An arrow represents a causal link between an SGA and a DEG, while the weight of an arrow represents the posterior probability that the SGA causes the DEG in the current tumor. *PIK3CA* is predicted to be the most probable cause for DEG *MPL* in 200 tumors; thus, we rank *PIK3CA* as 1^st^. *PTEN* is the most probable cause for DEG *MPL* in 140 tumors, ranking it as the 2^nd^ most probable cause of DEG *MPL*. *AKT1* is the most probable cause for DEG *MPL* in 11 tumors, and *PIK3R1* is the most probable cause for DEG *MPL* in 10 tumors. **C.** eQTL analysis of the possible causes of DEG *MPL*. eQTL considers all SGAs (i.e., ~17,600 SGAs) as possible causes for DEG *MPL*. The *p* values of *PIK3CA*, *PTEN*, *PIK3R1* and *AKT1* were ranked as having the 3^rd^, 5,690^th^, 7,661^th^ and 14,563^th^ strongest association with DEG *MPL*, respectively.

We applied TCI to analyze data from 5,097 tumors across 16 cancer types in TCGA (https://cancergenome.nih.gov/, S1 Table) to derive 5,097 tumor-specific models (one causal network model per tumor). As a concrete example to illustrate the characteristics of the TCI framework, we present the TCI results of discovering the tumor-specific causes of differential expression of the proto-onco-gene *MPL* that is commonly observed in tumors. Thrombopoietin receptor *MPL* (TPO-R), a major regulator of megakaryocytopoiesis and platelet formation, is a proto-oncogene whose ligand (TPO) has been recently identified as a novel candidate marker for ovarian cancer diagnosis and is associated with a poor survival [35, 36]. In recent work, Ismail el al. developed a breast cancer mouse model and found *MPL* is linked to cell death induction and tumor growth suppression [37]. There is evidence to indicate that the expression of *MPL* is regulated by the PI3K/AKT signaling pathway [38], which includes as members *PIK3CA*, *PTEN*, *PIK3R1*, *AKT1* (Fig 2A), and other gene products. The uniqueness of TCI lies at the fact that it seeks to learn the causal relationships between SGAs and DEGs at the individual tumor level. Our assumption is that a DEG is likely to be regulated by one aberrant pathway in a tumor, and such a pathway usually is perturbed by an SGA that affects one member of the pathway. This assumption is based on the observation that SGAs perturbing members of a common pathway rarely co-occur in an individual tumor, which is a phenomenon referred to as *mutual exclusivity* [39–41]. Thus, tumors with an aberrant PI3K/AKT pathway usually host an SGA in one of these members, although they likely share target genes, e.g., the DEG *MPL*. A causal discovery algorithm should attribute an *MPL* DEG event in a tumor to a member SGA of the PI3K/AKT pathway with high probability if one of those SGAs appears in the tumor. Indeed, in the tumors exhibiting differentially expressed *MPL*, TCI assigns the highest probability to a member of PI3K/AKT pathway if it is altered in the same tumor. TCI identified *PIK3CA* as the most frequent cause for DEG of *MPL* (in 200 tumors), while *PTEN* is ranked the second most common cause (in 140 tumors). Interestingly, *AKT1* and *PIK3R1* are also among the top 10 most frequent causes of an *MPL* DEG (in 11 tumors and 10 tumors, respectively) (Fig 2B).

As a comparison, we also performed eQTL analyses [33, 34] to identify the SGAs that are associated with DEG events in *MPL*. eQTL assesses the strength of association of genomic variations on a quantitative trait (e.g., expression of a gene) at the population level, which is often used to study functional consequence of genomic variations. To perform eQTL analysis, we used the R package MatrixEQTL [42] which is a widely used tool specifically designed for ultra-fast eQTL analysis of large datasets (589 citations since 2012). It is also an official tool of the GTEx project (https://gtexportal.org/home/) and is used in the seeQTL browser (https://seeqtl.org/). We evaluated the association of all SGAs observed in TCGA with respect to expression change of *MPL*, and the *p* values for the association of four members of the PI3K/AKT pathway were ranked as 3^rd^ (*PIK3CA*), 5,690^th^ (*PTEN*), 7,661^th^ (*AKT1*) and 14,563^th^ (*PIK3R1*) among all other SGA events (Fig 2C) observed in the TCGA PANCAN cohort. For each tumor, we identified the SGA that had the strongest association with *MPL* DEG, according to the eQTL-derived *p*-values. The results showed that *PIK3CA* was ranked 1^st^, *PTEN* was ranked 113^rd^, *PIK3R1* was ranked 128^th^, and *AKT1* was ranked 165^th^ as possible causes for *MPL* DEG in individual tumors. Thus, while eQTL analysis can identify *PIK3CA* as an important regulator of *MPL* expression, unlike TCI it does not attribute SGAs in other members of PI3K/AKT pathway as major causes for the changed expression of *MPL* at the population level.

### TCI predicts the most probable tumor-specific causative SGA for each DEG

We defined an SGA event in a tumor as an *SGA with functional impact* (SGA-FI) if it was predicted by TCI to causally regulate 5 or more DEGs in the tumor with an expected false discovery rate ~ 10^−7^ for discovering SGA-FIs from randomized *in silico* experiments, which is determined based a series of random simulation experiments. (Methods and S1 Fig).

We identified a total of 634 genes that were called as SGA-FIs in more than 30 tumors with an SGA-FI call rate of 25% or greater in our pan-cancer analysis (Methods and S2 Table). The call rate for an SGA *A*_*h*_ is the ratio of number of tumors in which *A*_*h*_ is designated an SGA-FI over the number of tumors in which *A*_*h*_ occurs. These SGA-FIs include the majority of the previously published drivers [1, 2], as well as many novel candidate drivers. For 302 well known drivers from literature [1, 2], we found 93 were called as significant SGA-FIs and 262 were called as SGA-FIs in at least one tumor. Note that if SGAs in a well-known driver do not affect gene expression, e.g., mutation or deletion of *BRCA1*, TCI would not be able to detect its functional impact. In addition to protein-coding genes, TCI also identified SGA events affecting microRNAs and intergenic non-protein-coding RNAs (e.g., *MIR31HG* [43, 44], *MIR30B* [45], and *PVT1* [46]) as SGA-FIs, (S2 Table).

We further identified target DEGs for the 634 significant SGA-FIs. To minimize false discovery, we required that a target DEG of an SGA-FI be regulated by the corresponding SGA-FI in at least 50 tumors or in 20% or more of all tumors in which the SGA was called as an SGA-FI. Since it is statistically difficult to evaluate whether the causal relationship between an SGA-FI and its predicted target DEG within an individual tumor is valid, we adopted a “pan-cancer” analysis approach to determine whether each predicted SGA→DEG causal relationship is conserved across tumors in different cancer types. We appreciate that there are cancer-type-specific effects, and we did applied the TCI algorithm to tumors of each tissue of origin or cell type to infer the causal relationships, although our presentation did not concentrate on such results. We addressed this issue in two major ways. First, when performing pan-cancer analysis, the goal is to identify the causal relationships that are shared among different cancer types, and conservation of causal relationships across different cancer types is a strong indication that the discovered causal relationship is more likely to be true. Therefore, we required that a causal edge is conserved in at least two types of cancers when TCI is applied to tissue-specific data. Second, since tissue-specific prevalence of certain SGAs and DEGs can create a confounding effect, in that they may appear to have correlations in a subset of tumors at the pan-cancer level. To mitigate such confounding effects, we specifically identified tissue-specific DEGs and removed them from pan-cancer analysis. We determine a DEG is a tissue-type-specific DEG if it exists in more than 90% of the tumors in one cancer type or tissue type while it appears in less than 1% of the tumors in other cancer types. We found and removed 44 such DEGs from further analysis (Materials and methods). We then set out to assess whether the inferred causal relationships are supported by existing knowledge and experimental studies. Finally, we performed preliminary laboratory experiments on selected SGA-FIs to evaluate the causal relationships between novel candidate drivers and their target DEGs predicted by TCI.

### The landscape of causative SGAs identified by TCI

**Fig 3:**
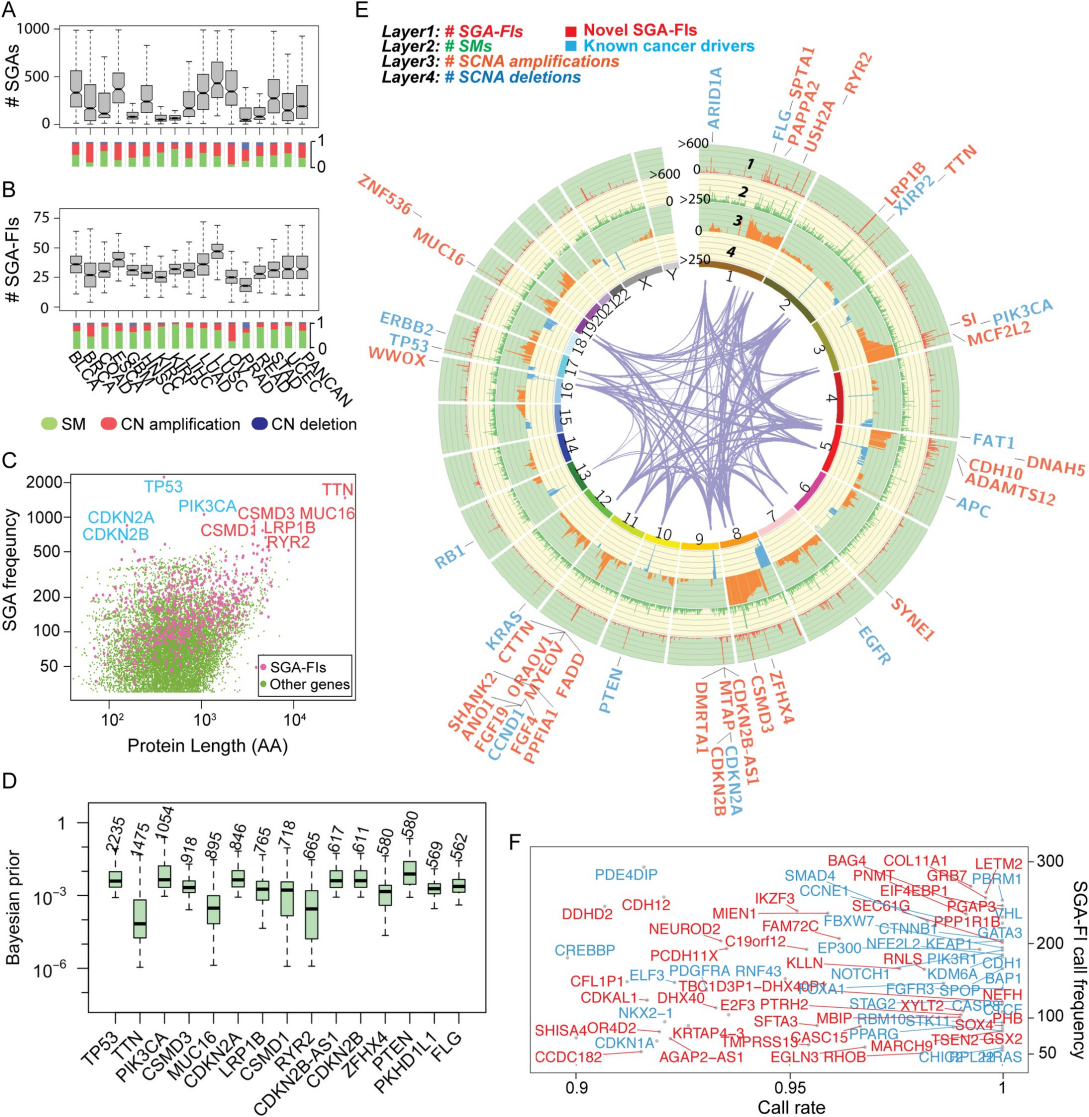
The landscape of SGAs and SGA-FIs. **A & B.** The distributions of SGAs per tumor and SGA-FIs per tumor of different cancer types. Beneath the bar box plots, the distributions of different types of SGAs (SM, copy number amplification, and deletion) are shown. **C.** Distribution of SGA-FIs against the alteration frequency and protein length. Pink dots indicate SGA-FIs, and green dots represent SGAs that were not designated as SGA-FIs. A few commonly altered genes are indicated by their gene names, where genes labeled with blue font are well-known drivers, and those labeled with orange font are novel candidate driver. **D.** Tumor-specific Bayesian prior distributions for top 15 most frequent SGAs. The number above each box represents number of tumors that the corresponding SGA appears in. **E.** A Circos plot shows SGA events and SGA-FI calls along the chromosomes. Different types of SGA events (SM, copy number amplification, deletion) are shown in tracks 2, 3, and 4, respectively. Track 1 shows the number of times that an SGA is labeled by TCI as an SGA-FI. The gene names denote the top 62 SGA-FIs (some are SGA units) that were called in over 300 tumors with a call rate > 0.8. Genes labeled with blue font are known drivers from two TCGA reports, and orange ones are novel candidate drivers. **F.** SGA-FIs that were called in less than 300 tumors and with a call rate > 0.9 are shown in this frequency-vs-call rate plot. As before, genes labeled with blue font are known drivers from TCGA studies, and orange ones are novel candidate drivers.

We compared the distribution of the number of SGAs and SGA-FIs per tumor across cancer types (Fig 3A and 3B). The average number of SGAs per tumor across cancer types was 268, whereas the average number of SGA-FIs identified by TCI was approximately 34 per tumor. Interestingly, TCI designated all SGAs with very high alteration frequency (perturbed in more than 500 tumors, or > 10%) as SGA-FIs (Fig 3C). One immediate concern for TCI is that it might call certain long genes, such as *TTN* and *MUC16*, as SGA-FIs solely due to their high genomic alteration rate. This concern was addressed by adopting statistical test results from MutSigCV analysis, which specifically addressed the biased mutation rate introduced by lengths and chromosome locations of genes. In our analysis, we represented the predicted effect of gene length and location by way of the prior probability term *P*(*A*_*h*_→*E*_*i*_), and as such, the prior probabilities for certain long genes, such as *TTN* and *MUC16*, were several orders of magnitude lower than other frequently altered well-known drivers (Fig 3D). Thus, the strength of statistical relationships between SGAs in these genes and their target DEGs, as conveyed by the marginal likelihood term *P*(*D*|*A*_*h*_→*E*_*i*_), must be sufficiently high to overcome the low prior probabilities of these genes being regulators of DEGs. Many SGA-FIs with an alteration frequency ranging from 30 to 500 tumors (0.5–10%) appear among other SGAs with similar protein lengths and alteration rates (Fig 3C). Since genes with similar protein length and alteration rate usually have similar prior probabilities of being drivers, TCI differentiated SGA-FIs from others based mainly on the difference in marginal probability *P*(*D*|*A*_*h*_→*E*_*i*_) associated with an SGA and candidate target DEGs. These results indicate that the function-oriented nature of TCI plays a significant role in detecting SGA-FIs.

We illustrated the landscape of common SGA events (Fig 3E) using a Circos plot (http://circos.ca/), and highlighted 44 SGA-FIs identified by TCI in more than 300 tumors (> 6% of the tumors) with a call rate (fraction of SGA instances affecting a gene being called as an SGA-FI event) greater than 0.8. The plot illustrates the integrative approach of TCI, which combines different types of SGA events in a gene and detects their function impact. For example, TCI combined mutation and deletion events in *LRP1B* (at 1 to 2 o’clock position on the plot) to detect common functional impact of these SGA events (see later section), whereas calling SGA-FI events for *ERBB2* (Her2) is mostly associated with amplification of the gene. TCI also designated many relatively low-frequency SGAs as SGA-FIs (in ~ 30 tumors or ~ 0.5%) with high call rates (> 0.9) (Fig 3F). Of interest, besides identifying well-known cancer drivers, e.g., *TP53*, *PIK3CA*, *PTEN*, *KRAS*, and *CDKN2A*) as SGA-FIs, TCI also designated as SGA-FIs some very frequently altered genes, e.g., *TTN*, *CSMD3*, *MUC16*, *LRP1B*, and *ZFHX4*, whose roles in cancer development remain controversial. These genes are excluded from driver gene lists when assessed by mutation-centered and frequency-based methods [2, 16], but other computational and experimental studies [47–49] suggest that some of them are likely cancer drivers.

### Combining SM and SCNA enhances detection of the functional impact of genes affected by SGAs

A cancer driver gene is often perturbed by multiple types of SGA events that exert common functional impact. For example, an oncogene, such as *PIK3CA*, is usually affected by activating mutations or copy number amplifications, whereas a tumor suppressor, such *PTEN*, is usually affected by inactivating mutations or copy number deletions. An SCNA event (amplification or deletion of a chromosome fragment) in a tumor often encloses many genes, making it a challenge to distinguish the functional impact of genes within a SCNA fragment.

**Fig 4:**
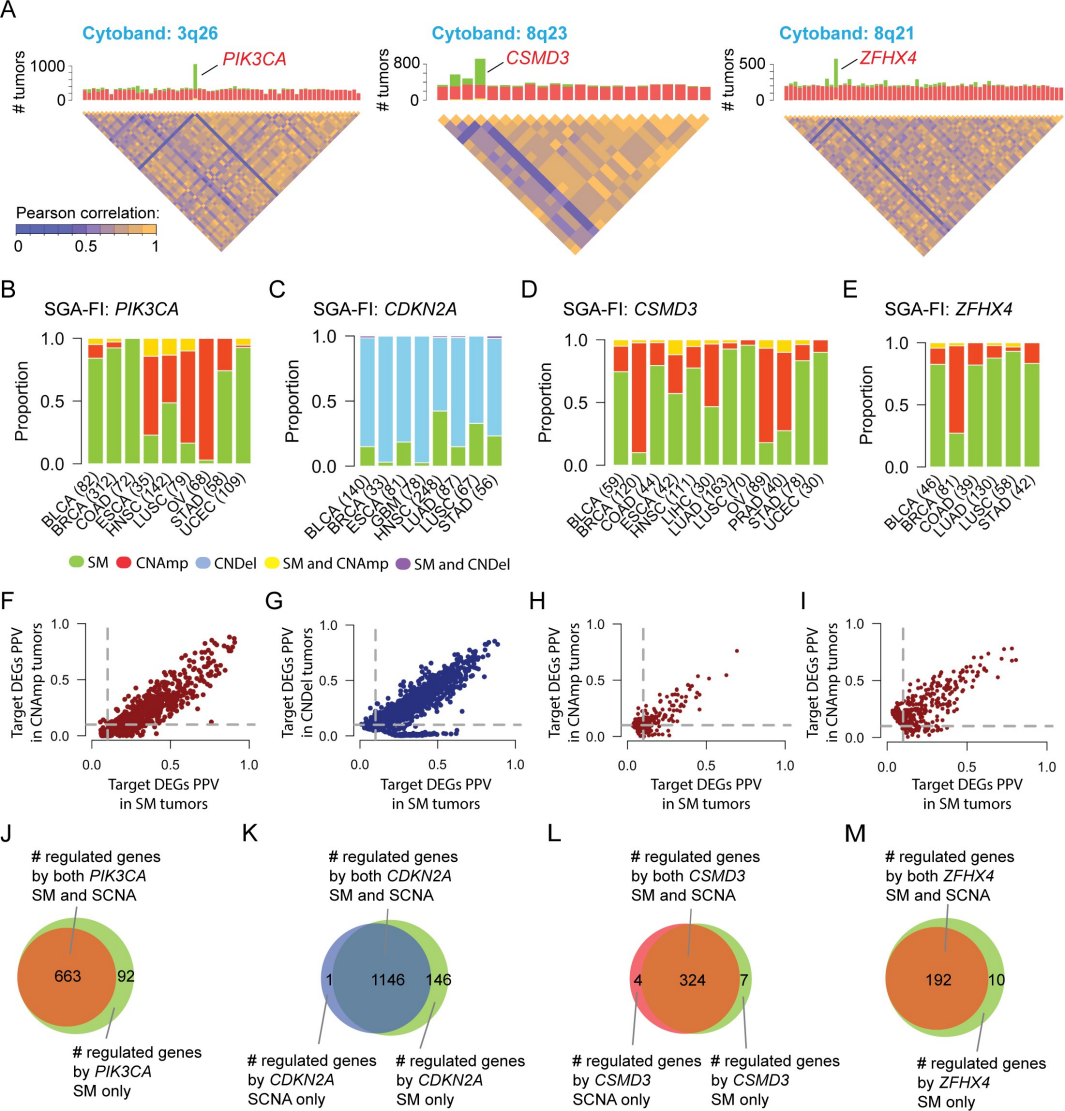
SM and SCNA perturbing a gene exert common functional impact. **A**. Combining SM and SCNA data drisrupts the correlation structure among genes enclosed in common SCNA fragments. The chromosome cytobands enclosing three example genes (*PIK3CA*, *CSMD3*, *and ZFHX4*) are shown. The bar charts show the frequency of SCNA (red, standing for amplificaton) and SM (green). The disequilibrium plots beneath the bar charts depict the correlationship among genes within a cytoband. **B-E.** The SGA patterns, i.e. SM and CN amplification/deletion, across different cancer types for *PIK3CA*, *CDKN2A*, *CSMD3* and *ZFHX4*, **F-I.** SGA-FI target DEG call rates in SM tumors and CN amplification/deletion for *PIK3CA*, *CDKN2A*, *CSMD3* and *ZFHX4*. **J-M.** Venn diagrams illustring the relationships of DEGs caused by CN amplication/deletion and SM for *PIK3CA*, *CDKN2A*, *CSMD3* and *ZFHX4*.

TCI addresses this problem by integrating both SM and SCNA data, which can create variances in overall SGA events among genes within a SCNA fragment. When combined with SM data, *PIK3CA* clearly has a higher combined alteration rate than its neighbor genes in the same DNA region with very similar amplification rate in cytoband 3q26. While its neighbor genes share almost identical copy number amplification profile across all tumors, the alteration profile of *PIK3CA* is significantly different when both SM and SCNA data are considered (Fig 4A). When calculating whether amplification of *PIK3CA* is causally responsible for a DEG observed in a tumor, TCI uses the statistics collected from all tumors with *PIK3CA* alterations, including both CN amplification and SM, to compute the marginal likelihood and predict whether a causal relationship between *PIK3CA* amplification and the DEG exists in the tumor. As such, the algorithm is able to differentiate the functional impact of *PIK3CA* amplification from that of other co-amplified genes. We noted that many genes were affected by both SMs and SCNAs patterns, including *CSMD3* and *ZFHX4* (Fig 4A), enabling TCI to detect the functional impact of these SCNA events.

By combining both SM and SCNA data, TCI is able to identify common functional impact of distinct types of SGA events affecting the same gene across different tumors and cancer types. For example, *PIK3CA* is often perturbed by either SMs or CN amplifications (Fig 4B) although prevalence of each type is different in different cancer types. In breast cancers (BRCA), *PIK3CA* is commonly altered by SMs; in ovarian cancers (OV), it is more often affected by CN amplification; in head and neck squamous carcinoma (HNSC), it is almost equally altered by SMs and CN amplification. As a well-known cancer driver in many cancer types, it is expected that amplification and mutations of *PIK3CA* should share a common functional impact in causally regulating a common set of DEGs.

Taking advantage of the tumor-specific inference capability of TCI analysis, we identified the target DEGs regulated by each SGA event affecting *PIK3CA* (either SM or SCNA) in individual tumors. DEGs predicted to be caused by either *PIK3CA* SM or CN amplification have very similar positive predictive values (PPV) with respect to SGA events in *PIK3CA*. The PPV is calculated as the ratio of number of tumors in which a DEG is designated as target of an SGA-FI such as *PIK3CA* over all tumors in which *PIK3CA* is called as an SGA-FI (Fig 4F and Methods), which reflect the strength of causal relationships between an SGA and its target DEG. The results indicate that perturbation of *PIK3CA* by both SM and CN amplification have very similar functional impact on gene expression changes. We then examined whether target DEGs caused by *PIK3CA* SM overlap with those caused by CN amplification, and indeed the DEG members of the two list significantly overlapped (Fig 4J). Thus, TCI detected the shared functional impact of distinct types of SGAs perturbing *PIK3CA* across different cancer types. Similar results were obtained for other 249 SGA-FIs (S3 Table) that were commonly perturbed by both SMs and SCNAs (with each type accounting for > 20% of instances for each SGA-FI), including *CDKN2A*, *CSMD3* and *ZFHX4* (Fig 4).

### Causal relationships inferred by TCI are statistically robust

**Fig 5:**
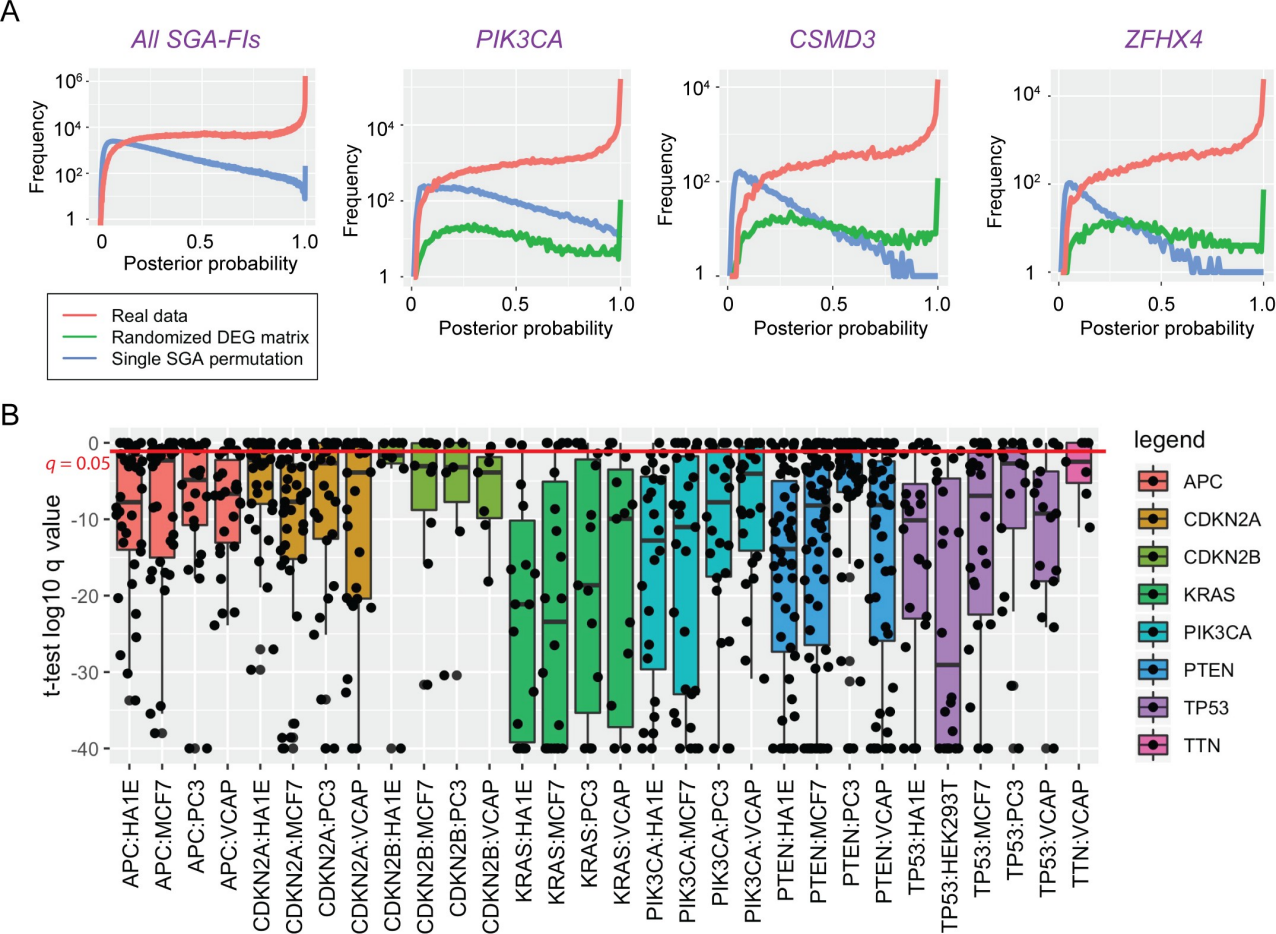
Statistical and experimental evaluation of TCI predictions. **A.** The causal relationship inferred by TCI is statistically sound. Plots in this panel show the probability density distribution of the highest posterior probabilities assigned to each DEG in TCGA dataset, when the TCI algorithms was applied to real data (red) and two random datasets, in which DEGs permutated across all tumors (blue) and the corresponding SGA permutated across all tumors (green). The panel on the left shows the results for the posterior probabilities for all most probable candidate edges in whole dataset; rest of the plots show the distributions of posterior probaiblities of most probable edges pointing from 3 specific SGAs to predicted target DEGs. **B.** Boxplots of *q-*values of t-test associated with predicted target DEGs for 8 SGA-FIs in different LINCS cell lines that were experimentally perturbed. Each box represent one SGA perturbed in one cell line. For example, APC-HA1E denotes that *APC* perturbed in HA1E cell line. Each black dot represents a *q-*value associated with a target DEG of an SGA-FI, when the expression value was assessed with a *t*-test of the before and after genetic manipulation of a given SGA-FI gene.

To evaluate validity of the results by TCI, we first examined whether the causal relationships reported by TCI reflect true statistical relationships between SGA and DEG events rather than random noise in the data. We generated a series of random datasets using the TCGA data, in which the DEG status of each gene expression variable was permuted among the tumors, while the SGA status in each tumor remained as reported by TCGA. After permutation, the statistical relationships between SGAs and DEGs are expected to be random. We then applied TCI to these random datasets and compared the posterior probabilities of the most probable causal edges for each DEG derived using real and permuted data. The results (Fig 5A) show TCI was able to differentiate true statistical relationships between SGAs and DEGs from random ones in that it assigned higher posterior probabilities to candidate edges obtained from real data (red lines) than those obtained from random data (blue lines). As expected, a large number of derived causal edges from well-known cancer drivers (e.g., *TP53* and *PIK3CA*) were assigned high posterior probabilities. Interestingly, the results also show many causal edges from other common SGA-FIs (*TTN*, *CSMD3*, *MUC16*, and *ZFHX4*) to DEGs were also assigned higher posterior probabilities than would be expected by random chance, indicating that perturbing these genes had significant impact on transcriptomics of the tumors (Fig 5A and S2A Fig). The function-oriented nature of TCI is reflected by observations that there are certain SGAs with a high alteration frequency (occurring in close to 10% of tumors) that were not designated as SGA-FIs by TCI. For example, *WASHC5* has SGA events in 457 tumors but few of these SGA events were assigned with high posterior probabilities of being SGA-FIs by TCI; similar results were observed for *TBC1D31* (424 tumors) and *ADGRB1* (420 tumors) (S2B Fig).

To further exclude the possibility that TCI-reported causal relationships from a high-frequency SGA to DEGs were random associations due to their high alteration frequencies, we conducted another series of single-SGA-permutation experiments, in which the SGA events of a gene (e.g., *TTN*) were randomly permuted across all tumors to disrupt the statistical relationships between SGAs of this gene and DEGs, while the overall frequency of the SGAs of the gene remains the same. We performed such single-SGA-permutation experiments for the 6 most commonly altered genes: *TP53*, *TTN*, *PIK3CA*, *CSMD3*, *MUC16*, and *ZFHX4*. The results (Fig 5A and S2B Fig) also show that when TCI analysis was applied to these permuted data (green lines), none of these 6 genes were designated as an SGA-FI according to our criteria. Taken together, these results support that TCI is detecting valid (non-spurious) statistical relationships between SGA and DEG events in real data.

### Causal relationships inferred by TCI are biologically sensible

We further evaluated whether the TCI-inferred causal relationships between SGAs and DEGs agree with existing knowledge and experimental results. We compared the predicted causal relationships between *PIK3CA* and DEGs with experimental results from an independent study. Recently, Hart et al. [50] studied the functional impact of a single mutation, H1047R, of *PIK3CA* by knocking in the mutation into the breast epithelial cell line MCF-10A and comparing the transcriptomic profile between the wild type and the *PIK3CA*^H1047R^ isogenic cell lines, which is the only transcriptomic study that is associated with the *PIK3CA* hotspot mutation so far. They identified 1,434 DEGs caused by the introduction of the mutation. We note that there exist differences among different cell lines and also between cell lines and tumor samples. In order to bridge the gap between cell lines and Pan-cancer tumor samples, we extracted the BRCA samples and compared the TCI-predicted *PIK3CA* target DEGs in breast cancer tumors with that from Hart’s study. We found that 12 out of 92 TCI-predicted *PIK3CA* SM driving DEGs overlap with the experimentally-derived DEG set (hypergeometric test *p* = 0.01).

Since RB1 protein regulates the function of transcription factor E2F1[51], it is expected that E2F1-regulated genes should be enriched among the *RB1*-targeted DEGs predicted by TCI. We used the PASTAA program[52] (trap.molgen.mpg.de/PASTAA.htm) to search for motif binding sites in the promoters of the 237 DEGs that TCI predicted to be regulated by *RB1*, and it found that *E2F1*, *E2F2*, and *DP-1* were the three top transcription factors for these genes (*p* < 10^−6^).

We also used the large-scale perturbation experiments carried by the Library of Integrated Network-Based Cellular Signatures (LINCS) project [53] to evaluate predicted causal relationships between SGA-FIs and their predicted target DEGs. The LINCS project performed systematic gene-manipulation (knockdown and overexpression) experiments using small interfering RNAs targeting over 4,000 genes in multiple cell lines, and cellular responses were measured as expression changes in 978 landmark genes (using a technology referred to as the L1000 assay). We selected the 8 most frequent SGA-FIs that were also experimentally manipulated in the LINCS project and performed *t*-tests on the expression values of all L1000 genes and analyzed the results of the perturbation experiments relative to the control condition in each cell line. We then examined the statistical significance of these differences and assessed the false discovery rate (*q* values) associated with the predicted target DEGs of each SGA. For each of the 8 SGA-FIs, the majority of predicted target DEGs were differentially expressed in multiple cell lines after experimental manipulation of the SGA-FI genes (Fig 5B). We note that certain target DEGs of an SGA have tissue-specific expression patterns, and we organized targeted DEGs according to tissue of origins and examined the percent of DEGs responding to manipulation of corresponding SGAs (S4 Table). Interestingly, we also found that *TTN* was perturbed in one cell line (VCAP), and 5 out of 7 predicted target DEGs responded to manipulation of *TTN*. Among them, 4 genes (*SPP1*, *STAT1*, *C5* and *GPER1*) are known to be associated with development and/or progression of cancer [54–57]. In summary, the causal relationships between SGAs and DEGs predicted by TCI were supported by multiple lines of examination, including the use of existing knowledge of these relationships as well as targeted and systematic experimental results.

**Fig 6:**
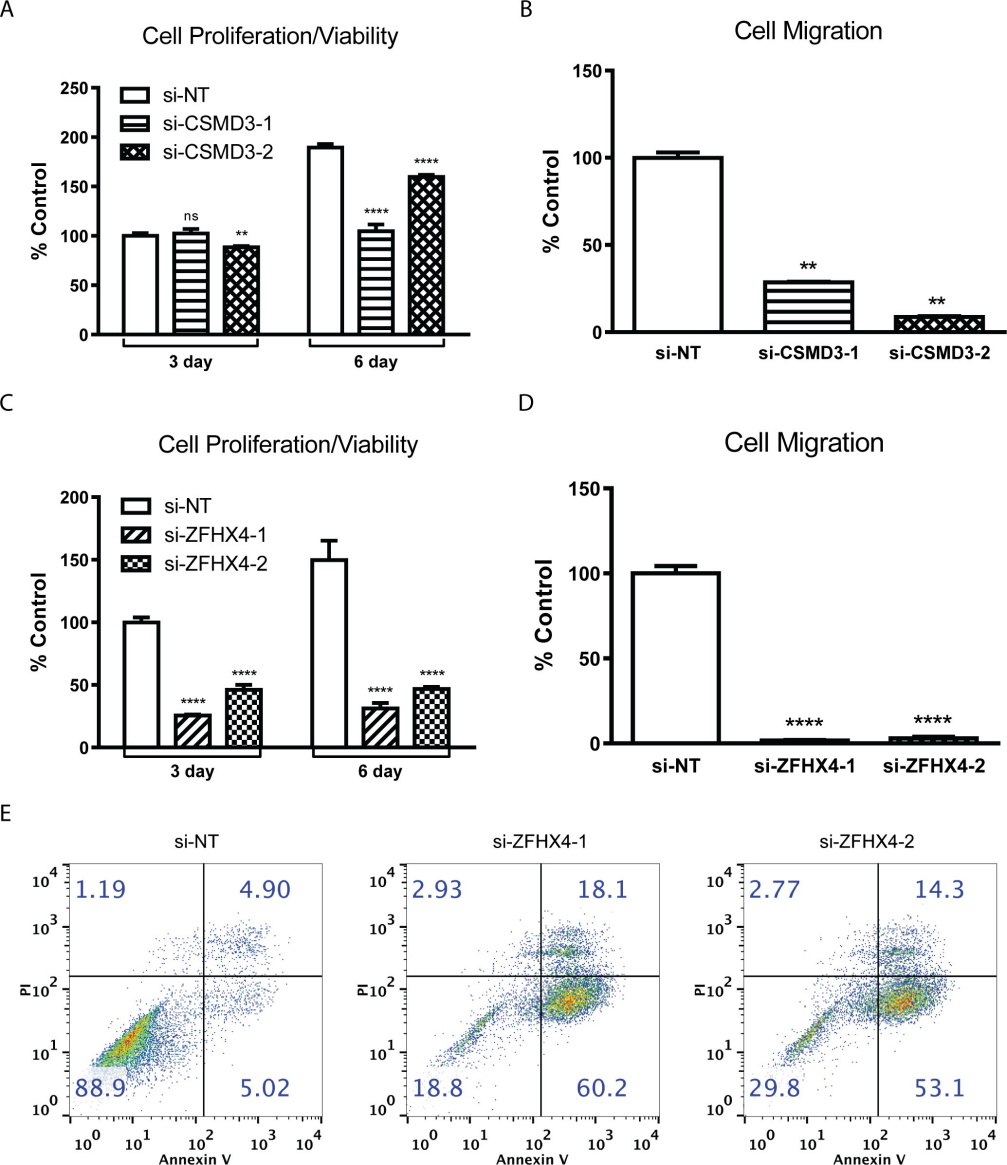
Cell biology evaluation of oncogenic properties of *CSMD3* and *ZHFX4*. **A-B**. The impact of knocking down *CSMD3* and *ZFHX4* on cell proliferation. **C-D**. The impact of knocking down *CSMD3* and *ZFHX4* on cell migration. **E**. Impact of *ZFHX4* knockdown on apoptosis in PC3 cell line measured by Annexin V and propidium iodide (PI) staining.

TCI indicated that *CSMD3* and *ZFHX4* are 4^th^ and 12^th^ most frequent SGA-FIs, and yet, they are designated as cancer drivers (S2 Table) in previous studies [17–22]. We examined whether experimental manipulations of *CSMD3* and *ZFHX4* expression affect oncogenic phenotypes. We identified two cancer cell lines, HGC27 and PC3, with *CSMD3* and *ZFHX4* amplification respectively, and we knocked down the expression of the two genes using siRNAs, followed by monitoring cellular phenotypes (see Methods for details). Our results showed that knocking down *CSMD3* and *ZFHX4* in the respective cell lines significantly attenuated cell proliferation (viability) and migration (Fig 6A–6D). In addition, knockdown of *ZFHX4* induced apoptosis (Fig 6E). These results provide support that these genes are involved in maintaining the cancer-related cellular phenotypes in these cell lines.

### SGA-FIs regulate genes involved in well-known oncogenic processes

**Fig 7:**
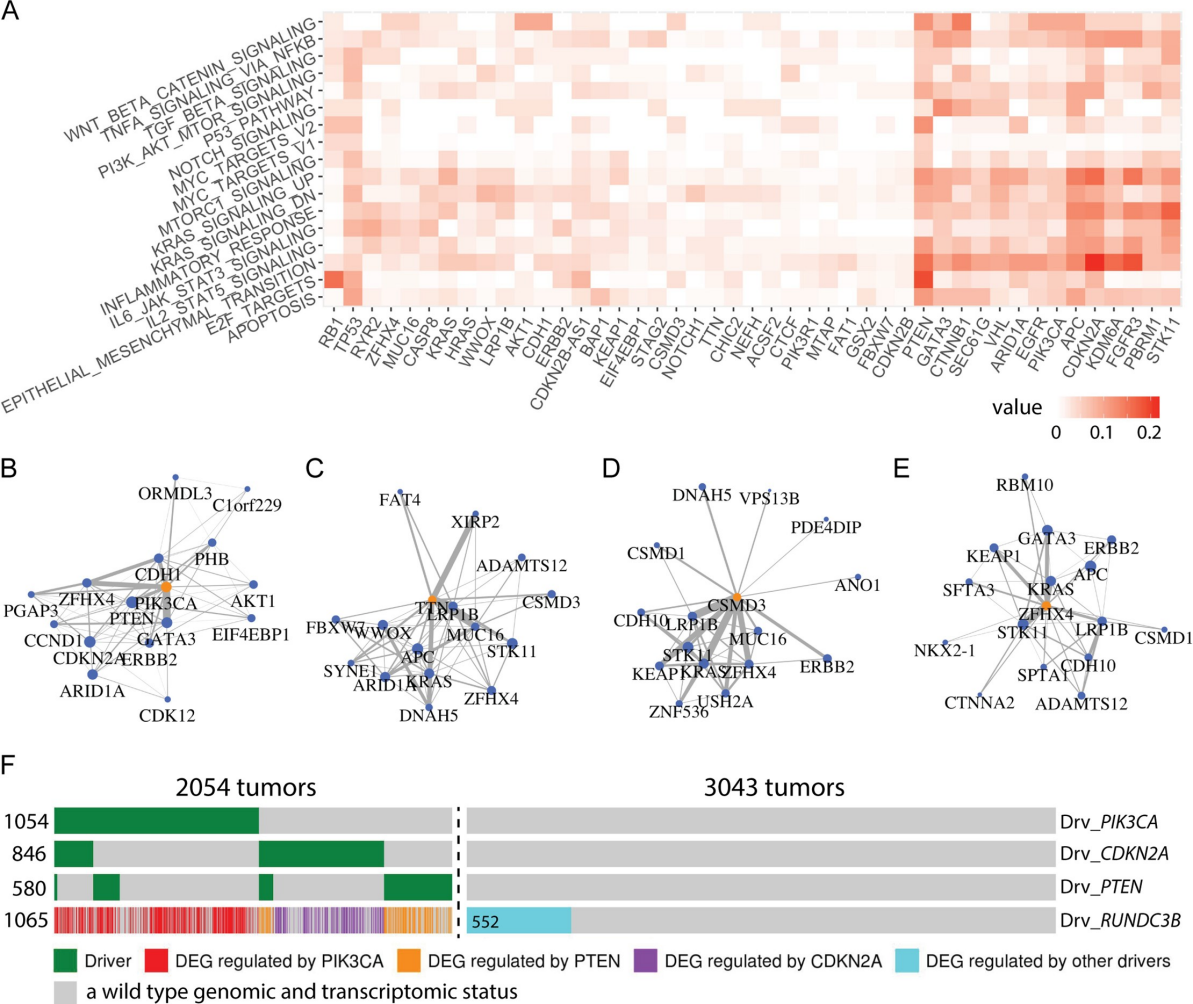
Detection of functional impact of SGA-FIs reveals functional connections among SGA-FIs. **A.** Top 45 SGAs-FIs (regulating the largest number of DEGs) and their relationships with 17 cancer hallmark gene sets. The value in a cell represents the fraction of genes in a hallmark gene set that is covered by the target DEGs of each SGA-FI. **B-E.** Top 15 SGA-FIs that share the most significant overlapping target DEGswith *PIK3CA*, *TTN*, *CSMD3*, and *ZFHX4*. An edge between a pair of SGA-FI indicate that they share significantly overlapping target DEG sets, and the thickness of the line is proportional to negative log of the *p*-values of overlapping target DEG sets. **F.** An “oncoprint” illustrating the causal relationships between the DEG *RUNDC3B* and its 3 main drivers according to TCI, namely, *PIK3CA*, *CDKN2A*, and *PTEN*. Each column corresponds to a tumor; green bars indicate tumors in which TCI designated each of the three SGA genes as a driver, regardless of what DEGs it was driving in a given tumor. The causal relationship is color-coded, which illustrates which SGA-FI is predicted by TCI to cause the *RUNDC3B* DEG event; the blue bar indicates the DEG events that were assigned to SGA-FIs other than the above 3 SGA-FIs; gray bars indicate a wild type genomic and transcriptomic status.

To gain a better view of functional impacts of SGA-FIs in cancer development, we further examined their impact on 1,855 genes from 17 cancer-related “hallmark” gene sets from the MSigDB (http://software.broadinstitute.org/gsea/msigdb/index.jsp). On average, 374 cancer hallmark genes are found to be differentially expressed in a tumor. TCI found 96 SGA-FIs that are predicted to regulate members of these 1,855 hallmark genes. These results illustrate the impact of an SGA on cancer hallmark processes. We listed the relationships between the 96 SGA-FIs with respect to the 17 cancer hallmark processes to identify the target DEGs for each of 96 SGA-FIs (S5 Table). The relationships between top 45 SGA-FIs with largest number of target DEGs with respect to the hallmark processes are shown in (Fig 7A). For example, *CTNNB1* is known as the top regulator of WNT pathway and it is predicted by TCI to cause 14% DEGs in HALLMARK_WNT_BETA_CATENIN_SIGNALING pathway [58]; *RB1* regulates 15% of the genes in HALLMARK_E2F_TARGETS [59]; *TP53* regulates genes involved in apoptosis and in a broad assortment of functions across many other oncogenic pathways [60, 61]; Our analysis also suggests that *CDKN2A* plays an important role in the epithelial-mesenchymal transition (EMT) process, which agrees with previous studies [62].

### TCI analyses reveal functional connections among SGA-FIs

The causal relationships between SGAs and DEGs revealed by TCI enable us to explore whether distinct SGAs in different tumors do in fact perturb a common signal, by examining if they share overlapping target DEGs. To this end, we evaluated all pair-wise intersections between target DEG sets of SGA-FIs to identify SGA pairs sharing significantly overlapping target DEGs (*p* < 0.05 Fisher’s exact test, and *q* < 0.05), and found 2669 such SGA-FI pairs (S6 Table). We then organized SGA-FIs that perturb common signals into a graph, in which an edge connecting a pair of SGA-FI nodes indicates significant overlap of their target DEGs. For example, the top 15 SGA-FIs (ranked according to the FDR *p* values of overlapping DEG sets) that share DEGs with *PIK3CA* include *PTEN*, *CDH1*, *ERBB2*, and *GATA3*, which are known cancer drivers, and their connections agree with existing knowledge (Fig 7B) [63–65].

The capability of revealing functional connections among SGAs provides a means of evaluating whether a novel candidate driver shares functional impact with well-known drivers, which not only provides an indication of whether the candidate driver is involved in oncogenic processes (and thus a candidate cancer driver gene) but also sheds light on which pathway it may be involved in. The top 15 SGA-FIs sharing common target DEGs with *TTN* include some well-known drivers including *APC*, *KRAS*, and *STK11* (Fig 7C). Therefore, *TTN* may share similar functional impact with these known drivers. The top 15 SGA-FIs connected with *CSMD3* and *ZFHX4* (Fig 7D and Fig 7E) also form densely connected networks that include well-known cancer drivers, such as *KRAS*, *GATA3*, *KEAP1*, *ERBB2* and *STK11*, suggesting that alteration of *CSMD3* and *ZFHX4* may perturb some of the same signaling pathways as do these known drivers. We found similar results for other common SGA-FIs, including *CDKN2A*, *PTEN*, *MUC16*, and *LRP1B* (S3 Fig).

Transcription of a gene is often regulated by a pathway, and it is expected that major driver SGAs of a DEG should include members of such a regulatory pathway. As an example, Fig 7F shows the SGA events that TCI designated as the cause of differential expression of *RUNDC3B* in different tumors. The TCI analysis indicates that *PIK3CA* is the most common cause. Besides SGAs in *PIK3CA*, TCI inferred that SGAs in *CDKN2A and PTEN* are two other major drivers of *RUNDC3B* DEG events. The results suggest that aberrations in PI3K pathway (as a result of SGAs perturbing *PIK3CA* and *PTEN*) is the main cause of these DEG events, and *CDKN2A* may act as an alternative regulator. It is also interesting to note that in certain tumors when both SGAs affecting *CDKN2A* and *PTEN* were present, TCI assigned *PTEN* as the most likely driver of *RUNDC3B*, instead of *CDKN2A*, even though the SGAs in the latter are more frequent. The results indicate that although *CDKN2A* SGA events explain the overall DEG variance of *RUNDC3B* better than *PTEN*, the strength of statistical association between *PTEN* and some DEGs in certain tumors may be stronger than that of *CDKN2A*, and TCI can detect such statistical relationships.

### Tumor-specific causal inference reveals tumor-specific disease mechanisms

TCI analysis enables us to identify major SGAs that causally regulate molecular phenotypic changes (in the current case, DEGs) in an individual tumor. In this way, TCI not only discovers potential drivers of an individual tumor but also suggests which oncogenic processes they may affect. Thus, TCI can provide insights about tumor-specific disease mechanisms, particularly when more oncogenic phenotypic data types become available, such metabolomic data and protein expression data.

**Fig 8:**
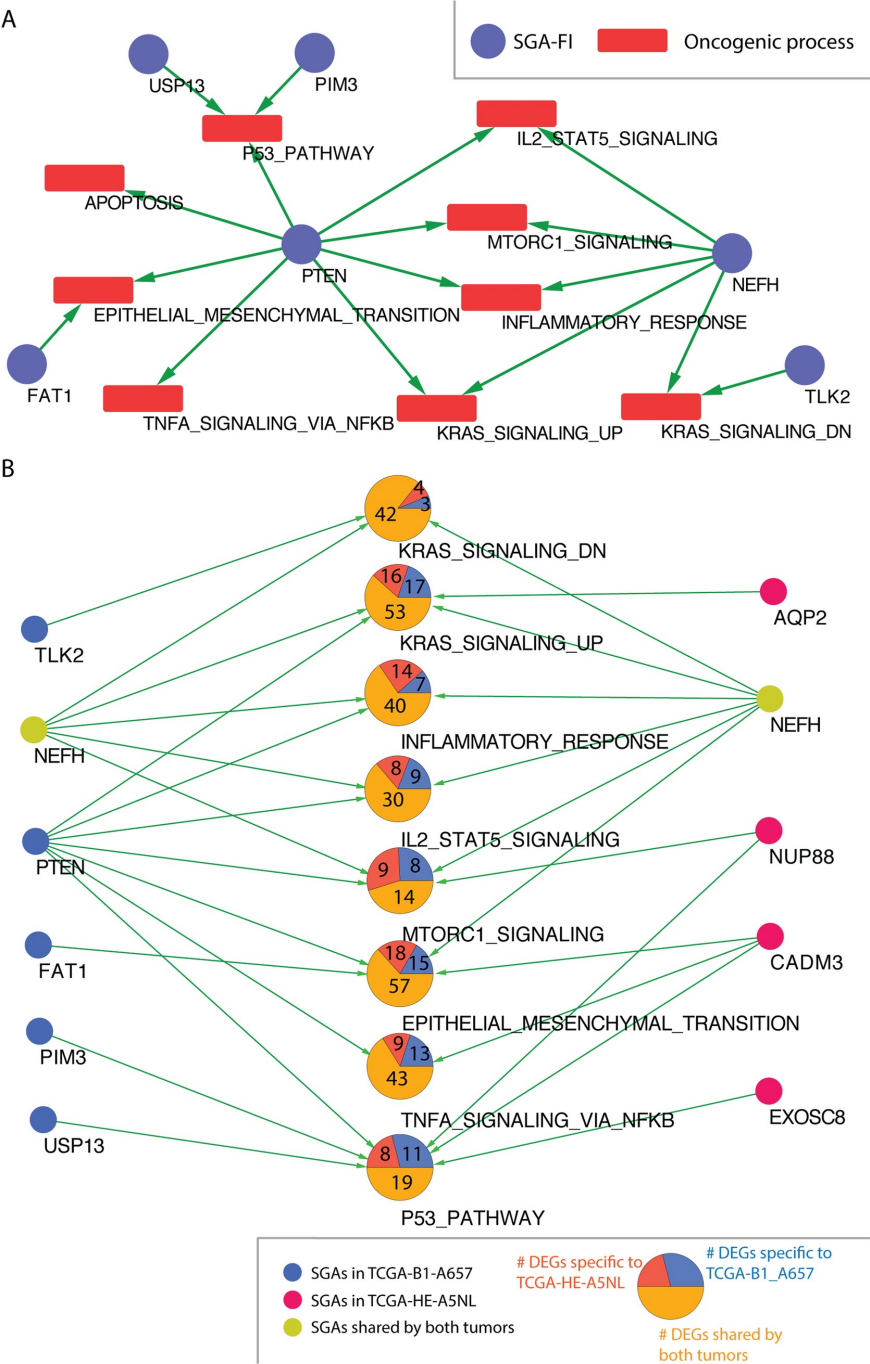
TCI predicts the SGA-FIs and their functional impact at the individual tumor level. **A.** A graph produced by TCI for tumor TCGA-B1-A657 that predicts major SGA-FIs and their regulated cancer processes. Blue nodes represent SGA-FIs and red nodes (squares) represent oncogenic processes. An green directed link indicates that TCI predicts that the SGA-FI at the tail of tha arrow regulates 10% or more of the DEGs in the cancer process at the head of the arrow. **B.** Same DEGs regulated by distinct SGA-FIs in different tumors. DEGs in cancer processes shared between tumor TCGA-B1-A657 and tumor TCGA- HE-A5NL are shown as pie-charts. Blue nodes denote SGA-FIs in tumor TCGA-B1-A657. Red nodes denote SGA-FIs in tumor TCGA- HE-A5NL. Yellow nodes, (i.e., *NEFH*), are shared by both tumors. Each large node in the middle represents an oncogenic process. Within the circular nodes in the middle of the figure, the number in the purple area denotes the number of DEGs specific to TCGA-B1-A657. The number in the red area denotes the number of DEGs specific to TCGA- HE-A5NL. The number in the yellow area denotes the number of DEGs shared by both tumors. An green directed link indicates an SGA-FI regulates 10% or more DEGs in the cancer process.

TCI results enabled us to examine each tumor profiled by TCGA to identify the major candidate driver SGAs and their target DEGs. Further examining the DEGs involved in hallmark biological processes allows us to study which biological processes an SGA affects. As an example, Fig 8A shows the SGA-FIs and their target cancer processes for a tumor (TCGA-B1-A657) of Kidney Renal Papillary cell carcinoma (KIRP), where genes in 9 oncogenic hallmark process from MSigDB are significantly enriched among the DEGs, including the following pathways that are strongly regulated by one of more SGA-FIs: the Epithelial Mesenchymal Transition pathway, the KRAS signaling pathway, the TNFA signaling via NFKB pathway, and the IL2 STAT5 signaling. We also identified major SGA-FIs (according to the number of DEGs regulated by them in the tumor) that affect these processes (Fig 8A). In this figure, a green arrow indicates that an SGA-FI regulates at least 10% of the genes in the corresponding signaling pathway. TCI identified 6 such SGA-FIs, including some well-known cancer drivers, such as *PTEN* and *NEFH*, and potential cancer drivers mentioned in recent studies, such as *TLK2*[66], *USP13*[67], and *PIM3*[68].

SGAs cause cancer by perturbing cellular signaling pathways, and a pathway usually consists of multiple signaling proteins. Thus, it is possible that tumors having very distinct SGA profiles may in fact share very similar patterns of pathway perturbation, thus sharing similar gene expression profiles. We further identified another KIRP tumor (TCGA-HE-A5NL), which shares a similar overall DEG profile to that of TCGA-B1-A657 (Fig 8B). These two tumors shared 281 DEGs related to the aforementioned oncogenic processes, and many DEGs in each oncogenic process were shared by the two tumors. However, each of these two tumors also had its unique SGA set, such as 57 SGAs in TCGA-B1-A657, 65 in TCGA-HE-A5NL, and only 2 common SGAs (*CADM3* and *NEFH*). TCI discovered similar target DEGs for *NEFH* in both tumors. Although many DEG members in each oncogenic process were shared, different SGA-FIs were designated as their candidate drivers. The above results illustrate that TCI is able to suggest disease mechanisms of individual tumors, and such information can be further analyzed to suggest tumors sharing common disease mechanisms.

## Discussion

TCI is novel computational framework to assess whether a genomic alteration event causally influences one or more molecular/cellular phenotypes at the level of individual tumor. This tumor-specific and causality-center framework provides a new perspective to study cancer driver genes and disease mechanisms of individual tumors. The tumor-specific nature of TCI enables discovering causal relationships and shedding light on disease mechanism of an individual tumor. Further exploring the commonality and differences in disease mechanisms of a large number of tumors in the population will significantly help us better understand cancer biology in general. More importantly, understanding the disease mechanism of each tumor lays a solid foundation for guiding personalized therapies and advancing precision oncology. The causality-centered nature of the TCI provides a unifying framework to combining data (statistics) of different types of SGAs, eliminating the need of separately assessing whether mutations, or SCNAs, or other SGA events in a gene are over-enriched in a cancer population by conventional approaches, which would require reconciling measurements and baseline models associated with each type of SGA. Integrating diverse types of SGA events is statistically sensible which increases the statistical power for assessing the functional impact of perturbing a candidate driver gene. It is also biologically sensible that a driver gene is often perturbed by different types of SGA events leading to common functional impact. The fact that a gene is often perturbed by different types of SGAs leading to common phenotypic changes provides strong support that the gene is a candidate driver because its functional impact is positively selected in cancer.

Our analyses of TCGA data revealed the functional impact of many well-known, as well as a large number of novel SGA-FIs, with a wide range of prevalence in tumors ranging from 1% to more than 10%. These results serve as a catalogue of major SGA events that potentially contribute to cancer development. Discovery of novel candidate drivers also provides potential targets for developing new anti-cancer drugs. By revealing the functional impact of candidate drivers (e.g., a signature of DEGs), TCI results can be utilized to identify SGAs sharing similar functional impact and to discover cancer pathways *de novo* or to map novel candidate drivers to known pathways.

Interestingly, TCI revealed functional impact of certain SGAs with very high alteration frequencies, such as *TTN*, *CSMD3*, *MUC16*, *RYR2*, *LRP1B*, and *ZFHX4*, whose roles in cancer development remain controversial. There are studies indicating that their high mutation rates are likely due to heterogeneous mutation rates at different chromosome locations [2, 16]. TCI analysis provides a new perspective to examine the role of these genes: assessing whether perturbations (considering all SGA events) in these genes are supported as causally influencing molecular and cellular phenotype changes. Instead of concentrating on assessing whether its frequency is above random chance, TCI evaluates the functional impact of an altered gene that determines whether it contributes to (drives) cancer development. Our results suggest that perturbing these genes, either by genome alterations, such as SM and/or SCNA, or by experimental manipulations, has significant impact on molecular and cellular changes in both tumors and cell lines. Therefore, these results motivate further investigation of an alternative hypothesis for high overall alteration rates of these genes in cancer: perturbation of these genes leads in a variety of ways to functional changes that provide oncogenic advantages. The results suggest that utilizing diverse types of SGA events in these genes is in fact a result of positive selection.

The TCI model can be extended in several ways. First, with its capability of integrating heterogeneous data types, TCI can be further extended to include additional SGA types (e.g., DNA methylation) and molecular phenotypes (e.g., protein expression and metabolomics data) in order to provide a more comprehensive model of the causal relationships within tumor cells. Such extensions can be readily achieved by representing such events in the SGA data matrix, with minimum change in the TCI algorithm. Second, the functional impact of each SGA (i.e., either activating or repressing the gene expression) should be further studied to determine the SGA-FI as an oncogene or a tumor suppressor. Third, the TCI search algorithm can be relaxed to allow synergistic interactions between SGAs in regulating a single DEG which can be crucial to induce complex changes in gene expression pattern [69]. Last but not the least, the recent emergence of single cell multi-omics sequencing technology has enabled researchers to analyze gene mutations, copy number variants, methylations and gene expression changes simultaneously at the individual cell level [70–72]. When large, multi-omics, tumor single-cell cohort datasets become available, they will provide us the opportunity to perform TCI on tumor multi-omics data at the single cell level and advance our understanding of cell-to-cell variability and thus cancer progression.

### Conclusion

This paper presented the TCI algorithm, which concentrates on addressing a fundamental question in discovering cancer-driving genes: whether perturbation of a gene (considering different types of perturbations) is causally responsible for certain molecular/cellular phenotypes (considering different phenotypic measurements) relevant to cancer development in a tumor. We combined multiple heterogeneous genome data types and applied the TCI algorithm to 5,097 tumors across 16 cancer types from TCGA. TCI identified over 600 significant SGA-FIs, including many known drivers, which were further supported by our computational analysis and experimental evaluations. We illustrated that these SGA-FIs regulated expression changes of genes involved in well-known oncogenic processes. We showed that two tumor samples with very similar DEG expression profiles may nonetheless have significantly different SGA-FIs that account for those profiles. Thus, TCI provides a new statistical framework for predicting causal SGAs and understanding their functional impact on oncogenic processes of an individual tumor. Finally, TCI is a special case of a general instance-based causal inference framework [73, 74] that can be broadly used to delineate causal relationships between genomic variance and phenotype changes at the level of individuals which can be a single cell or an individual patient.

## Materials and methods

### SGA data collection and preprocessing

We obtained SM data for 16 cancer types directly from the TCGA portal (https://tcga-data.nci.nih.gov/tcga/dataAccessMatrix.htm) (accessed in October 2014). We considered all the non-synonymous mutation events of all genes and considered the mutation events at the gene level, where a mutated gene is defined as one that contains one or more non-synonymous mutations or indels.

SCNA data were obtained from the Firehose browser of the Broad Institute (http://gdac.broadinstitute.org/). TCGA network employed GISTIC 2.0[25] to process SCNA data, which discretized the gene SCNA into 5 different levels: homozygous deletion, single copy deletion, diploid normal copy, low copy number amplification, and high copy number amplification. We only included genes with homozygous deletion or high copy number amplification for further analysis. We further screened out the genes with inconsistent copy number alteration across tumors in a given cancer type (i.e., gene was perturbed by both copy number amplification and deletion events in the same cancer type and both types of events occurred > 25% of tumors).

We combined preprocessed SM data and SCNA data as SGA data, such that a gene in a given tumor was designated as altered if it was affected by either an SM event and/or an SCNA event.

### DEG data collection and preprocessing

Gene expression data were preprocessed and obtained from the Firehose browser of the Broad Institute. We used RNASeqV2 for cancer types with expression measurements in normal tissues. For cancer types without RNASeqV2 measurements in normal cells (i.e., glioblastoma multiforme and ovarian cancer), we used microarray data to identify DEGs. We determined whether a gene is differentially expressed by comparing the gene expression in the tumor cell against that in the corresponding tissue-specific normal cells. For a given cancer type, assuming the expression of each gene (log 2 based) follows Gaussian distribution in normal cells, we calculated the *p* values of each gene in a tumor, which estimated how significantly different the gene expression in tumor was from that in normal cells. If the *p* value was equal or smaller than 0.005 to either side, the gene was considered as differentially expressed in the corresponding tumor. Furthermore, if a DEG was associated with the SCNA event affecting it, we removed it from the DEG list of the tumor. We also removed tissue-specific DEGs if they were highly correlated with cancer types or tissue origin (i.e., Pearson correlation coefficient larger than 0.9). We thus identified the DEGs for each tumor and created a tumor-gene binary matrix where 1 represents expression change, and 0 represents no expression change.

### Tumor-specific model priors

Defining an informative prior that can represent the biological foundations of different genome alterations in tumor cells can help us effectively correct model bias and thus make accurate predictions [30, 75]. Therefore, we need to specify the model prior *P*(*A*_*h*_→*E*_*i*_) for each SGA *A*_*h*_ in each tumor *t* by comparing its alteration frequency in the tumor cohort against normal cells. In our paper, we used additional genomic information for both SM and SCNA to derive the prior probability of each edge *A*_*h*_→*E*_*i*_ using existing prior knowledge. We calculated and collected the following SGA information for each gene *h*: (1) the MutSigCV *p* value for *h* among the tumors in *D* from TCGA, and (2) the copy number amplification and deletion of *h* in a normal population without cancer from 1000 genome project (http://www.internationalgenome.org/) [76, 77]. Such information can be applied to help account for mutation and copy number alterations that are due to differences in gene lengths and chromosome locations which doesn’t depend on SGA frequency.

For a tumor *t* and an arbitrary DEG *E*_*i*_, we defined the prior probability of *A*_*h*_ being a parent of *E*_*i*_ using a multinomial distribution with a parameter vector *θ* = (*θ*_0_,*θ*_1_,*θ*_2_,…,*θ*_*h*_,…,*θ*_*m*_)^T^, where $\sum_{h=0}^{m}\theta_{h}=1$. Here, *θ*_0_ is a user-defined parameter representing the prior belief that the non-SGA factor *A*_*0*_ being the cause of *E*_*i*_, and *θ*_*h*_ represents the prior probability of *A*_*h*_ being the cause of *E*_*i*_. In this study, we set *θ*_0_ = 0.1. We assumed that *θ*_*t*_~*Dir*(*θ*_*t*_|*μ*_*t*_), where $\mu_{t}={\left(\mu_{0},\mu_{1},\ldots ,\mu_{h},\ldots ,\mu_{m_{t}}\right)}^{T}$ is a tumor-specific Dirichlet parameter vector governing the distribution of *θ*_*t*_. For a tumor *t*, we calculated the prior probability *θ*_*h*_ as follows:

$$\theta_{h}=(1-\theta_{0})\frac{\mu_{h}}{\sum_{h^{\prime }=1}^{m}\mu_{h}^{\prime }}$$ (3)

where *h’* indexes over the *m* variables in *SGA_SET*_*t*_; *p*_*h*_ is MutSigCV *p* value for *A*_*h*_ and *μ*_*h*_ = 1−*p*_*h*_ is a Dirichlet parameter.

We also analyzed three different ways of calculating *θ*_*h*_. First, as a simple default, we assume there are no informative priors. We distribute the residual probability mass evenly for all SGAs in tumor *t* as $\theta_{h}=(1-\theta_{0})\frac{1}{m}$. Second, we infer informative priors by incorporating SGA frequency as $\theta_{h}=(1-\theta_{0})\frac{f_{h}}{\sum_{h^{\prime }=1}^{m}f_{h}^{\prime }}$, where *f*_*h*_ is the alteration frequency of SGA *h*. The idea is that the driver genes should be positively selected to drive cancer progression, and therefore more likely are enriched in the tumor population. Third, we consider both SGA frequency and number of SGAs in each tumor so that the prior is calculated as $\theta_{h}=(1-\theta_{0})\frac{w_{h}}{\sum_{h^{\prime }=1}^{m}w_{h}^{\prime }}$, where $w_{h}=\sum_{t\in U_{h}}\frac{1}{m_{t}}$, *m*_*t*_ is the number of SGAs in tumor *t* and *U*_*h*_ denotes the tumor set in which SGA *h* has a genome alteration.

Different ways of calculating priors correspond to different biological assumptions, and thus, have distinct values, as shown in S7 Table. However, as illustrated in S8 and S9 Tables, a significant portion of the SGA regulators for DEGs and SGA-FIs called in each tumor remain the same for different priors. Thus, the strength of statistical relationships between SGAs and their target DEGs, as influenced by the marginal likelihood term *P*(*D*|*A*_*h*_→*E*_*i*_), are sufficiently high to overcome the differences in prior probabilities of some SGAs being regulators of DEGs even if the priors are calculated differently.

### Sensitivity analysis of A0 prior effect

We performed sensitivity analysis to examine the effect of the A0 prior. We used TCI to predict the SGA regulators for DEGs in each tumor using different A0 priors, i.e., 0.001, 0.005, 0.01, 0.05, 0.1, 0.2, 0.3, 0.4, 0.5. We compared the top SGA changes for DEGs in each tumor. As shown in S4 Fig, the top SGA changes are not significant, i.e. <0.08%, even though there is a 500-fold change for the A0 priors. We found TDI results are quite stable when the A0 prior is in the range of 0.05 to 0.3. We then set 0.1 as the prior probability for A0.

### Identification of SGA-FIs

Causal edges from different SGAs have different posterior probabilities, as expected. To standardize how to interpret the significance of a posterior probability for a causal edge *P*_*e*_, we designed a statistical test based random permutation experiments. We generated a series of permuted datasets using the TCGA data, in which the DEG values were permuted among the tumors of a common tissue of origin, while the SGA status in each tumor remained as reported by TCGA. This permutation operation disrupts the statistical relationships between SGAs and DEGs while retaining the tissue-specific patterns of SGAs and DEGs. We applied TCI algorithm to permuted data to calculate posterior probabilities of edges emitting from each SGA in random data. We then determined the probability that an edge from an SGA could be assigned with a given *P*_*e*_ or higher in data from permutation experiments (i.e., the *p* value to the edge with a given *P*_*e*_).

The *p* value in this setting is also the expected rate of false discovery of an SGA as the cause of a DEG by random chance. We utilize this property to control the false discovery rate when identifying SGA-FIs in a tumor. We designated an SGA event in a tumor as an SGA-FI if it has 5 or more causal edges to DEGs that are each assigned a *p-*value < 0.05. The overall false discovery rate of the joint causal relationships between an SGA to 5 or more target DEGs is smaller than 10^−7^. The S1 Fig shows that at this threshold, none of SGA was assigned as SGA-FI by random chance.

### Cell culture and siRNA transfection

HGC27 (Sigma-Aldrich) and PC-3 (ATCC) cells were cultured according to the manufacturer’s recommendations. The non-targeting and the *CSMD3* and *ZFHX4* siRNAs were obtained from OriGene (Rockville, MD). The siRNA sequences are as follow: si-CSMD3-1, GGUAUAUUACGAAGAAUUGCAGAGT; si-CSMD3-2, ACAAAUGGAGGAAUACUAACAACAG; si-ZFHX4-1, CGAUGCUUCAGAAACAAAGGAAGAC; si-ZFHX4-2, GGAACGACAGAGAAAUAAAGAUUCA. The siRNAs were transfected into cells using DharmaFECT transfection reagents for 48 hrs according to the manufacturer’s instructions.

### Cell proliferation and viability assays

Cell proliferation/viability was assayed by CCK-8 assay (Dojindo Laboratories, Kumamoto, Japan). Briefly, HGC27 and PC3 cells were plated at a density of 3 x 10^3^ cells/well in 96-well plates. After siRNA transfection for 3 or 6 days, CCK-8 solution containing a highly water-soluble tetrazolium salt WST-8 [2-(2-methoxy-4-nitrophenyl)-3-(4-nitrophenyl)-5-(2,4-disulfophenyl)-*2H*-tetrazolium, monosodium salt] was added to cells in each well, followed by incubation for 1–4 h. Cell viability was determined by measuring the O.D. at 450 nm. Percent over control was calculated as a measure of cell viability.

### Transwell migration assay

Cell migration was measured using 24-well transwell chambers with 8 μm pore polycarbonate membranes (Corning, Corning, NY). SiRNA-transfected cells were seeded at a density of 7.5 x 10^4^ cells/ml to the upper chamber of the transwell chambers in 0.5 ml growth media with 0.1% FBS. The lower chamber contained 0.9 ml of growth medium with 20% FBS as chemoattractant media. After 20 hrs of culture, the cells in the upper chamber that did not migrate were gently wiped away with a cotton swab, the cells that had moved to the lower surface of the membrane were stained with crystal violet and counted from five random fields under a light microscope.

### Apoptotic assay

Apoptosis was assessed by flow cytometry analysis of annexin V and propidium iodide (PI) double stained cells using Vybrant Apoptosis Assay Kit (Thermo Fisher Scientific, Carlsbad, CA). Briefly, the cells after washing with PBS were incubated in annexin V/PI labeling solution at room temperature for 10 min, then analyzed in the BD FACSCalibur flow cytometer (Becton, Dickinson and Company, Franklin Lakes, NJ).

## Supporting information

S1 Text Supporting information containing a detailed description of tumor-specific causal inference model. (DOCX)

S1 Table Number of tumors per cancer type collected from TCGA. (XLSX)

S2 Table S2.1. TCI predicted 634 Candidate SGA-Fis and their target DEGs; S2.2. Cancer type distribution of 634 Candidate SGA-FIs. (XLSX)

S3 Table SGA-FIs that are commonly altered by both SM and SCNA. (XLSX)

S4 Table Number of target L1000 genes for 8 most frequent SGA-FIs that are differentially expressed in different tissue types. (XLSX)

S5 Table Percentage of genes involved in the cancer Hallmark processes regulated by SGA-FIs. (XLSX)

S6 Table SGA-FI pairs sharing common target genes. (XLSX)

S7 Table Euclidean distance of log10 fold changes between different ways of calculating priors. (XLSX)

S8 Table Overlap ratio of top SGA regulator for DEGs in each tumor between different ways of calculating priors. (XLSX)

S9 Table Overlap ratio of SGA-FI calls in each tumor between different ways of calculating priors. (XLSX)

S1 Fig Controlling false discovery. **A.** The plot shows the relationship of total number of SGAs being designated as SGA-FIs with respect to the threshold of calling an SGA-FI in random and real data. The x-axis shows the different thresholds, i.e., the number of DEGs predicted to be regulated by an SGA-FI, and the y-axis shows the number of significant SGA-FIs across all tumors. **B.** The plot shows the relationship of average number of SGAs being designated as SGA-FIs in a tumor with respect to the threshold of calling an SGA-FI in random and real data. The x-axis shows the different thresholds, i.e., the number of DEGs predicted to be regulated by an SGA-FI, and the y-axis shows the average number of significant SGA-FIs in a single tumor.
(TIF)

S2 Fig Comparison of causal analysis results from real data and random data. **A.** Comparison of distributions of the posterior probabilities of the highest candidate causal edges point from 3 most frequent SGAs to DEGs. **B.** Examples of 3 genes with high SGA frequency but without any high posterior probability causal edges emitting from them. **C.** Comparison of number of tumors called as SGA-FIs from the real dataset, randomly permutated DEG dataset and single SGA permutated dataset for the 6 most frequency SGAs.
(TIF)

S3 Fig Networks of SGA-FIs share significant overlapping DEGs. **A.** SGA-FIs interacting network containing 536 SGA-FIs and 2669 edges. Blue nodes represent known cancer drivers and red nodes represent novel SGA-FIs. Node size indicates the number of its affected DEGs and edge width indicates the number of overlapped DEGs between two nodes. **B-E.** Top 15 SGA-FIs that share the most significant overlapping target DEGs with *CDKN2A*, *PTEN*, *LRP1B*, and *MUC16*. An edge between a pair of SGA-FI indicates that they share significantly overlapping target DEG sets, and the thickness of the line is proportional to negative log of the p-values of overlapping target DEG sets.
(TIF)

S4 Fig Top SGA change rate with respect to different A0 priors. (TIF)
