# Supplementary material for: Systematic discovery of the functional impact of somatic genome alterations in individual tumors through tumor-specific causal inference
Source: PLoS Comput Biol. 2019 Jul 5;15(7):e1007088. doi: 10.1371/journal.pcbi.1007088 (PMC6650088; doi:10.1371/journal.pcbi.1007088)
Supplement: S1 Text — (DOCX) [file pcbi.1007088.s001.docx]

Tumor-specific Causal Inference model

We designed the TCI algorithm to infer the functional-impact relationships between SGAs and DEGs for a given tumor using a bipartite causal Bayesian network (CBN) in which causal edges are only allowed to point from SGAs to DEGs. We assumed that each DEG is likely regulated by one aberrant pathway in an individual tumor and that such a pathway is likely perturbed by a single SGA due to the well-known mutual exclusivity among SGAs perturbing a common pathway (32-34).

Let **T** = {*T_1_*, *T_2_*, …, *T_t_*, …, *T_N_*} denote the tumor set that contains *N* tumor samples, where *t* indexes over the tumors included in the tumor set. Let *SGA_SET_t_* = {*A_1_*, *A_2_*, …, *A_h_*, …, *A_m_*} denote a subset of *m* genes with genome alterations in tumor *t* (i.e., the SGAs), where *h* indexes over the variables in *SGA_SET_t_*. Let *DEG_SET_t_* = {*E_1_*, *E_2_*, …, *E_i_*, …, *E_n_*} denote *n* genes that are differentially expressed in tumor *t*, where *i* indexes over the variables in *DEG_SET_t_* (i.e., the DEGs). We further included a variable *A_0_* to collectively represent factors other than SGAs (e.g., tumor microenvironment or hypoxia) that may cause differential gene expression.

For a given tumor *t*, a model *M* is a bipartite CBN in which, for each variable *E_i_* in *DEG_SET_t_*, there is exactly one arc into *E_i_* from a variable in *SGA_SET_t_*. In model *M*, a given *A_h_* can have zero, one, or more arcs emanating from it to the variables in *DEG_SET_t_*. Within the tumor *t*, TCI searches for a model *M* that best explains the data by assigning the *A_h_* in *SGA_SET_t_* that is the most probable cause for each *E_i_* in *DEG_SET_t_* . Pseudocode for the TCI algorithm is given below. Also shown is pseudocode for the algorithm FindGlobalDrivers, which produces results used by TCI, and thus must be called prior to calling TCI.

**FindGlobalDrivers**(*D*: dataset): set;

let *SGA_SET* be the set of all SEGs across all tumors

let *DEG_SET* be the set of all DEGs across all tumors

let *G* be an array of global drivers, one for each DEG variable

//a global driver of a DEG is the single best SGA that predicts that DEG over all tumors

for *E_i_* ∊ *DEG_SET* do

*bestScore* := 0;

for *A_h_* ∊ *SGA_SET* do

*score* := *e_global_*(*h*, *i*); //see Equation 5 below

if *score* > *bestScore* then

*bestSGA* := *A_h_*;

*bestScore* := *score*;

end for;

*G*(*E_i_*).*bestGlobalSGA* := *bestSGA*;

*G*(*E_i_*).*bestGlobalScore* := *bestScore*;

end for;

return *G*;

**TCI**(*t*: tumor; *D*: dataset; *G*: array of global drivers): CBN;

let *M* be a CBN;

initialize *M* to contain no arcs;

*SGA_SET_t_* := SGAs in tumor *t*;

*DEG_SET_t_* := DEGs in tumor *t*;

for *E_i_* ∊ *DEG_SET_t_* do

*bestScore* := 0;

for *A_h_* ∊ *SGA_SET_t_* do

*score* :=*e_local_*(*h*, *i*); //see Equation 8 below

if *score* > *bestScore* then

*bestSGA* := *A_h_*;

*bestScore* := *score*;

end for;

place the arc *bestSGA* 🡪 *E_i_* into *M*;

end for;

return *M*;

TCI employs a Bayesian framework to infer causal inference. In a tumor *t*, TCI scores an arc *A_h_* 🡪 *E_i_* between SGA *A_h_* and DEG *E_i_* based on the posterior probability of the arc, which can be derived as follows using Bayes rule:

$P\left( A_{h}\to E_{i} | D_{hi} \right)=\frac{P\left( D_{hi} | A_{h}\to E_{i} \right)P(A_{h}\to E_{i})}{\sum_{A\in{SGA\_SET}_{t}} P\left( D_{hi} | A\to E_{i} \right)P(A\to E_{i})}$, (1)

where $D_{hi}$ is the data about variables *A_h_* and *E_i_*; $P\left( D_{hi} | A_{h}\to E_{i} \right)$ is the marginal likelihood of the data given that *A_h_* is modeled as the cause of *E_i_*; and$P(A_{h}\to E_{i})$ is the prior probability that *A_h_* is the cause of *E_i_*. Since the posterior probability shown on the left side of Equation 1 is proportional to the numerator on the right side, TCI simply uses the numerator as the arc score. In the text below, we discuss how to derive the numerator in both a global and tumor-specific manner.

The *tumor-specific* nature of TCI is reflected in the following points: 1) Each tumor has a unique *SGA_SET_t_* and the *DEG_SET_t_*; thus, the TCI-inferred CBN structure *M* is tumor-specific. 2) The prior probability of a model $P(M)$ [which is based on the component priors $P(A_{h}\to E_{i})$] is tumor-specific. 3) Calculation of the marginal likelihood of an arc$P(D_{hi}|A_{h}\to E_{i})$ consists of two components: one component is computed using the data from the tumors in which *A_h_* =1, (i.e., “tumors like me”), and the other component is derived using a global model. As such, the posterior probability of the edge $A_{h}\to E_{i}$ is specific to a given tumor, and the same edge $A_{h}\to E_{i}$ may have different posterior probabilities in different tumors depending on the composition of *SGA_SET_t_.*

***Tumor-specific model priors.*** While it would be ideal to define the prior probability for each edge $P(A_{h}\to E_{i})$ using prior knowledge, we usually have very limited information about it. Despite of the lack of specific prior knowledge, we use the prior probability to incorporate the following general knowledge (on which frequency-oriented approaches are based) regarding the probability that a gene is a driver: the more frequently a gene is perturbed in a cancer cohort, the more likely it is a driver in an individual tumor. Furthermore, we also consider the tumor-specific context of the SGAs in each tumor.

To define an informative prior that can represent the biological foundations of different genome alterations in the tumor cells can help us effectively correct model bias and thus make accurate predictions (29,35). Therefore, we need to specify the model prior $P(A_{h}\to E_{i})$ for each SGA *A_h_* in each tumor *t* by comparing its alteration frequency in the tumor cohort against normal cells. In our paper, we used additional genomic information for both SM and SCNA to derive the prior probability of each edge $A_{h}\to E_{i}$ using existing prior knowledge. We calculated and collected the following SGA information for each gene *h*: (1) the MutSigCV *p* value for *h* among the tumors in *D* from TCGA, and (2) the copy number amplification and deletion of *h* in a normal population without cancer from 1000 genome project (http://www.internationalgenome.org/) (64,65). Such information can be applied to help account for mutation and copy number alterations that are due to differences in gene lengths and chromosome locations which doesn’t depend on SGA frequency.

For a tumor *t* and an arbitrary DEG *E_i_*, we defined the prior probability of *A_h_* being a parent of *E_i_* using a multinomial distribution with a parameter vector $\theta={(\theta_{0},\theta_{1}, \theta_{2},{\ldots,\theta}_{h},\ldots,\theta_{m} )}^{T}$, where $\sum_{h=0}^{m} \theta_{h}=1$. Here, *θ*_0_ is a user-defined parameter representing the prior belief that the non-SGA factor *A_0_* being the cause of *E_i_*, and $\theta_{h}$ represents the prior probability of *A_h_* being the cause of *E_i_*. In this study, we set $\theta_{0}$ = 0.1. We assumed that $\theta_{t}\sim Dir\left( \theta_{t} | \mu_{t} \right),$where $\mu_{t}=\left( \mu_{0},\mu_{1}, \ldots, \mu_{h},\ldots,\mu_{m_{t}} \right)^{T}$ is a tumor-specific Dirichlet parameter vector governing the distribution of $\theta_{t}$. For a tumor *t*, we calculated the prior probability $\theta_{h}$ as follows:

$\theta_{h}=(1-\theta_{0})\frac{\mu_{h}}{\sum_{h^{'}=1}^{m} \mu_{h}^{'}}$ (2)

where *h’* indexes over the *m* variables in *SGA_SET_t_*; $\mu_{h}=1-p_{h}$ is a Dirichlet parameter and $p_{h}$ is MutSigCV *p* value for *A_h_*.

***Marginal likelihood function P(D|M)***. In TCI, the overall marginal likelihood of the data *D* given model *M* is a product of the marginal likelihood of each arc` in *M*. The term $P\left( D | M \right)$ is the marginal likelihood function of *M*, which can be derived by marginalizing out model parameters $\theta$ as follows:

$$P\left( D | M \right)= \int_{\theta} P\left( D | M,\theta\right)P(\theta|M)d\theta\left( 3 \right)$$

where $\theta$ represents the parameters (probabilities) associated with causal Bayesian network structure *M*. Since our data (*A* and *E*) are discrete variables, the parameters and their prior distributions are multinomial and Dirichlet respectively. In addition, TCI uses the Bayesian Dirichlet equivalent uniform (BDeu)(66) scoring measure to derive the marginal likelihood for each arc in *M* as a function that is expressed in terms of the products and ratios of gamma functions. Taken together, we have the following:

$$P\left( D | M \right)= \prod_{i=1, n} P(D_{hi}|A_{h}\to E_{i})$$

$=\prod_{i=1}^{n} \prod_{j=1}^{q_{i}} \frac{\Gamma(\alpha_{ij})}{\Gamma(\alpha_{ij}+N_{ij})}\prod_{k=1}^{r_{i}} \frac{\Gamma(\alpha_{ijk}+N_{ijk})}{\Gamma(\alpha_{ijk})}$, (4)

where *j* indexes over the states of the cause of *E_i_* in *M* (i.e., some variable *A_h_*); *q_i_* is the number of possible values of *A_h_* (in our case, it is 2, because *A_h_* is modeled as a binary variable); *k* is variable which indexes over the states of *E_i_*; *r_i_* denotes the total possible states of *E_i_* (in our case, it is set to 2); *N_ijk_* is the number of tumors in dataset *D* in which node *E_i_* has value *k* and its cause *A_h_* in *M* has the value denoted by *j*; *α_ijk_* is a parameter in a Dirichlet distribution that represents prior belief about *P*(*E_i_* | *cause*(*E_i_*)); Γ is the gamma function; $N_{ij}=\sum_{k}^{r_{i}} N_{ijk}$; and $\alpha_{ij}=\sum_{k}^{r_{i}} \alpha_{ijk}$.

Let $e_{global}(h,i)$ represent the function that calculates the Bayesian score of the edge $A_{h}\to E_{i}$ in tumor *t* (i.e., the numerator of the Eq (1)) over all the data. Then, $e(h,i)$ can be defined as follows:

$e_{global}\left( h,i \right)= \theta_{G(i)}\prod_{j=1}^{q_{i}} \frac{\Gamma(\alpha_{ij})}{\Gamma(\alpha_{ij}+N_{ij})}\prod_{k=1}^{r_{i}} \frac{\Gamma(\alpha_{ijk}+N_{ijk})}{\Gamma(\alpha_{ijk})}$ . (5)

where $\theta_{G(i)}$ can be calculated using Equation (2).

The tumor-specific calculation of the marginal likelihood uses the data that are most relevant to the given tumor (aka “tumors like me”) to infer if the hypothesis of a candidate causal edge $A_{h}\to E_{i}$ is supported among these tumors. To do so, we modified the Bayesian scoring function shown in Equation 5 by dividing the training data *D* into a tumor-specific subset (the subset of tumors with *A_h_* = 1 for the current tumor *t*) and the remaining data (the subset of tumors with *A_h_* = 0). Let $D_{h}^{1}$ denote the subset of tumors in which *A_h_*=1 and $D_{h}^{0}$ denote the subset of tumors in which *A_h_*=0, such that $D=\left\{ D^{0}\cup D^{1} \right\}$. Let *G*(*i*) represent the SGA with maximal Bayesian score for *E_i_* derived from Equation 5 at the whole cohort level (referred to as “global driver”) and let $\theta_{G(i)}$ denote the prior probability that *G*(*i*) is the cause of *E_i_* in tumor *t*.

We can calculate the $e\left( h,i,D_{h}^{1} \right)$ and $e\left( G(i),i,D_{h}^{0} \right)$ for tumors with *A_h_*=1 and those with *A_h_*=0, respectively, as follows:

${e\left( h,i,D_{h}^{1} \right)=\theta}_{h}\prod_{j=1}^{q_{i}} \frac{\Gamma\left( \alpha_{ij} \right)}{\Gamma\left( \alpha_{ij}+N_{ij}^{1} \right)}\prod_{k=1}^{r_{i}} \frac{\Gamma\left( \alpha_{ijk}+N_{ijk}^{1} \right)}{\Gamma\left( \alpha_{ijk} \right)}$ (6)

${e\left( G\left( i \right),i,D_{h}^{0} \right)=\theta}_{G(i)}\prod_{j=1}^{q_{i}} \frac{\Gamma\left( \alpha_{ij} \right)}{\Gamma\left( \alpha_{ij}+N_{ij}^{0} \right)}\prod_{k=1}^{r_{i}} \frac{\Gamma\left( \alpha_{ijk}+N_{ijk}^{0} \right)}{\Gamma\left( \alpha_{ijk} \right)}$ (7)

$e_{tumor-specific}\left( h,i \right)= e\left( h,i,D_{h}^{1} \right)*e\left( G\left( i \right),i,D_{h}^{0} \right)$ (8)

where $N_{ijk}^{1}$ is the number of tumors in $D_{h}^{1}$ in which *E_i_* has value *k* and *A_h_* = 1; $N_{ijk}^{0}$ is the number of tumors in $D_{h}^{0}$ that *E_i_* has value *k*; and *A_G(i)_* has the value indexed by *j*. Finally, the posterior probability of a causal edge $A_{h}\to E_{i}$ can be calculated with the following equation:

$P\left( A_{h}\to E_{i} | D,{SGA\_SET}_{t},{DEG\_SET}_{t} \right)=\frac{e_{tumor-specific}(h,i)}{\sum_{h^{'}=0}^{m} e_{tumor-specific}(h^{'},i)}$ (9)
