# Supplementary figures and images for: Systematic discovery of the functional impact of somatic genome alterations in individual tumors through tumor-specific causal inference
Source: PLoS Comput Biol. 2019 Jul 5;15(7):e1007088. doi: 10.1371/journal.pcbi.1007088 (PMC6650088; doi:10.1371/journal.pcbi.1007088)

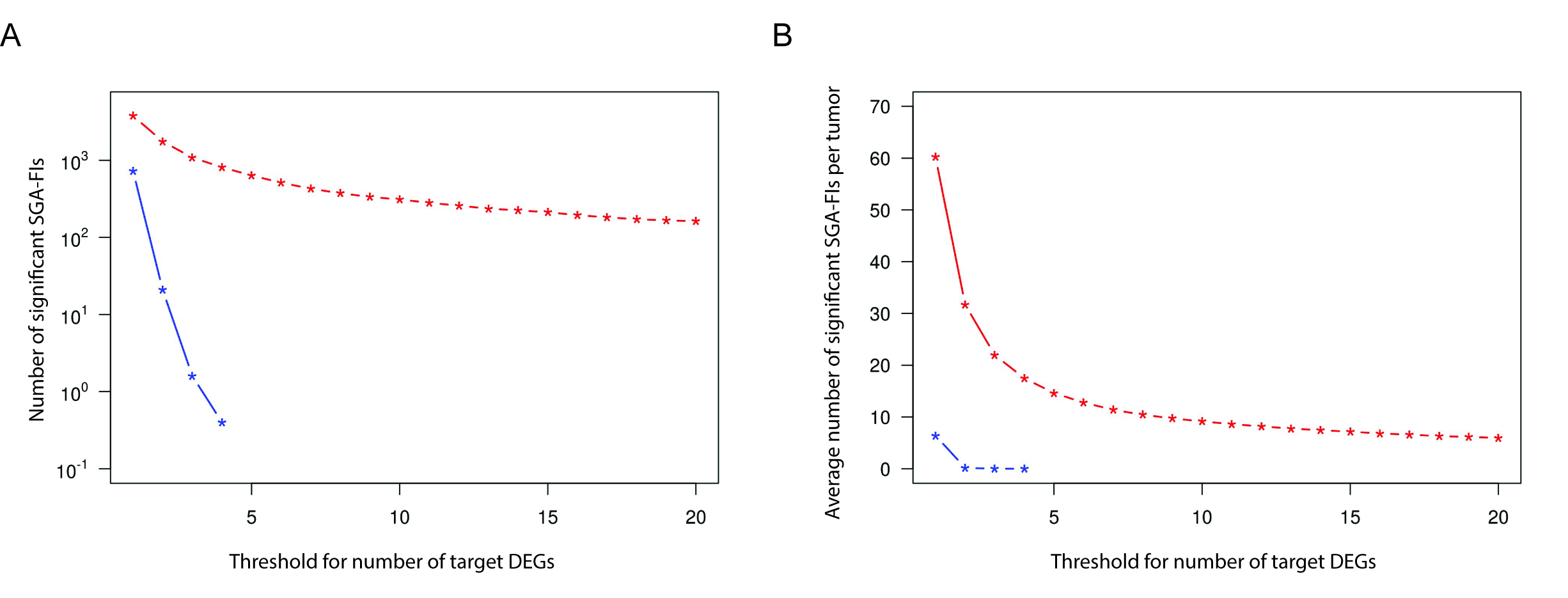

Supplement: S1 Fig — A. The plot shows the relationship of total number of SGAs being designated as SGA-FIs with respect to the threshold of calling an SGA-FI in random and real data. The x-axis shows the different thresholds, i.e., the number of DEGs predicted to be regulated by an SGA-FI, and the y-axis shows the number of significant SGA-FIs across all tumors. B. The plot shows the relationship of average number of SGAs being designated as SGA-FIs in a tumor with respect to the threshold of calling an SGA-FI in random and real data. The x-axis shows the different thresholds, i.e., the number of DEGs predicted to be regulated by an SGA-FI, and the y-axis shows the average number of significant SGA-FIs in a single tumor. (TIF) [file pcbi.1007088.s011.tif]

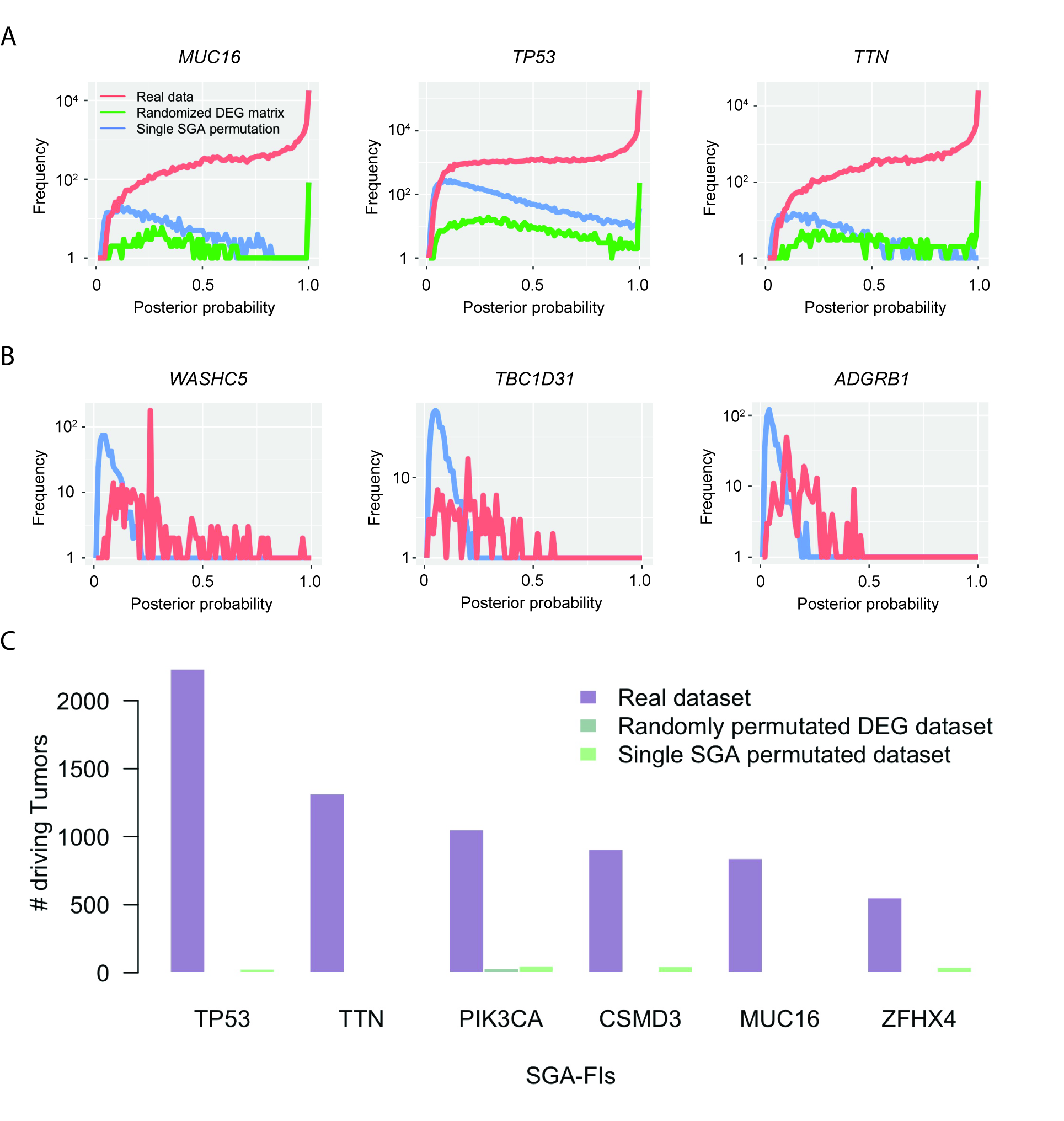

Supplement: S2 Fig — A. Comparison of distributions of the posterior probabilities of the highest candidate causal edges point from 3 most frequent SGAs to DEGs. B. Examples of 3 genes with high SGA frequency but without any high posterior probability causal edges emitting from them. C. Comparison of number of tumors called as SGA-FIs from the real dataset, randomly permutated DEG dataset and single SGA permutated dataset for the 6 most frequency SGAs. (TIF) [file pcbi.1007088.s012.tif]

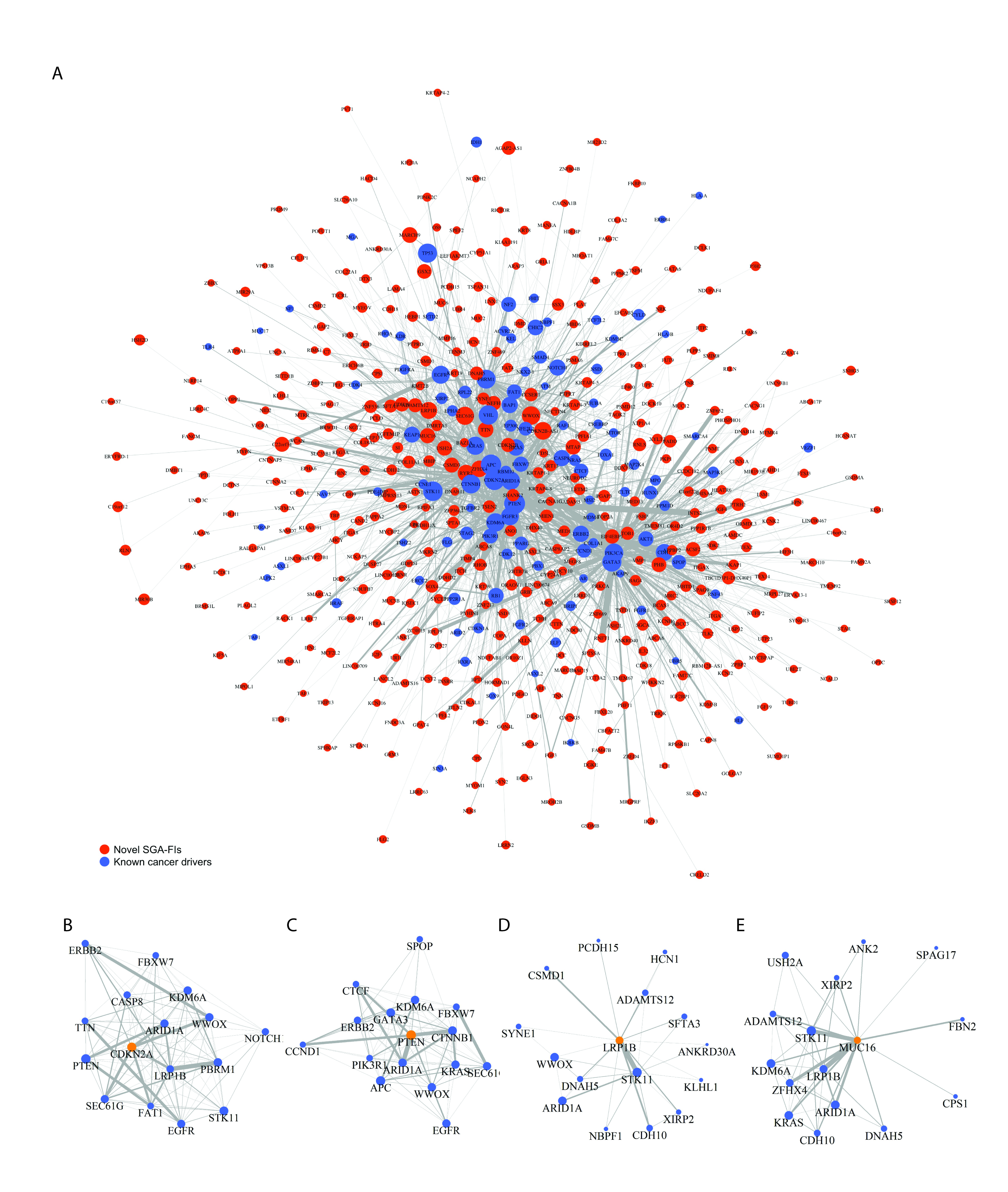

Supplement: S3 Fig — A. SGA-FIs interacting network containing 536 SGA-FIs and 2669 edges. Blue nodes represent known cancer drivers and red nodes represent novel SGA-FIs. Node size indicates the number of its affected DEGs and edge width indicates the number of overlapped DEGs between two nodes. B-E. Top 15 SGA-FIs that share the most significant overlapping target DEGs with CDKN2A, PTEN, LRP1B, and MUC16. An edge between a pair of SGA-FI indicates that they share significantly overlapping target DEG sets, and the thickness of the line is proportional to negative log of the p-values of overlapping target DEG sets. (TIF) [file pcbi.1007088.s013.tif]

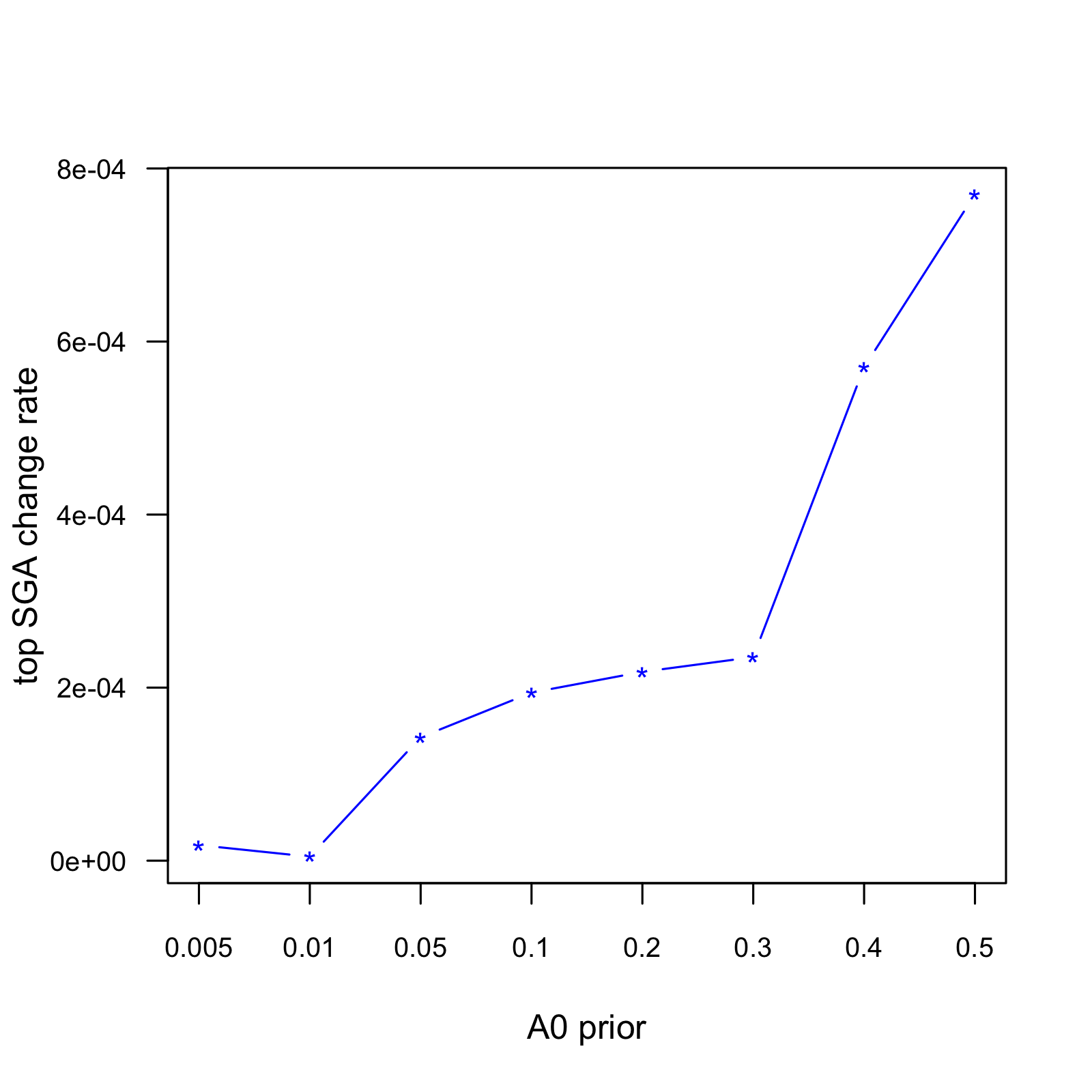

Supplement: S4 Fig — (TIF) [file pcbi.1007088.s014.tif]
